# Factors Associated with Health Inequalities in Infectious Disease Pandemics Predating COVID-19 in the United States: A Systematic Review

**DOI:** 10.1089/heq.2021.0049

**Published:** 2022-03-24

**Authors:** Karli K. Kondo, Beth E. Williams, Chelsea K. Ayers, Devan Kansagara, Mia Smith, Shailesh M. Advani, Sarah Young, Somnath Saha

**Affiliations:** ^1^ Evidence Synthesis Program, Veterans Affairs Portland Health Care System, Portland, Oregon, USA.; ^2^ Department of Research Integrity, Oregon Health & Science University, Portland, Oregon, USA.; ^3^ Early Cancer Detection Science, American Cancer Society, Kennesaw, Georgia, USA.; ^4^ Department of Primary Care, Veterans Affairs Portland Health Care System, Portland, Oregon, USA.; ^5^ OHSU-PSU School of Public Health, Portland, Oregon, USA.; ^6^ Department of Oncology, Georgetown University, Washington, District of Columbia, USA.; ^7^ Center to Improve Veteran Involvement in Care, Veterans Affairs Portland Health Care System, Portland, Oregon, USA.; ^8^ Division of General Internal Medicine and Geriatrics, Oregon Health & Science University, Portland, Oregon, USA.

**Keywords:** racial minorities, minority health, health disparities, COVID, low income, pandemic

## Abstract

**Objective:**

Previous pandemics may offer evidence on mediating factors that contributed to disparities in infection and poor outcomes, which could inform the effort to mitigate potential unequal outcomes during the current COVID-19 pandemic. This systematic review sought to examine those factors.

**Methods:**

We searched MEDLINE, PsycINFO, and Cochrane to May 2020. We included studies examining health disparities in adult U.S. populations during infectious disease epidemics or pandemics. Two investigators screened abstracts and full text. We assessed study quality using the Newcastle/Ottawa Scale or the Critical Appraisal Skills Programme Checklist for Qualitative Studies.

**Results:**

Sixteen articles were included, of which 14 focused on health disparities during the 2009 H1N1 influenza pandemic. Studies showed that disparities during the H1N1 pandemic were more related to differential exposure to the virus than to susceptibility or access to care. Overall, pandemic-related disparities emanate primarily from inequalities in social conditions that place racial and ethnic minorities and low socioeconomic status populations at greater risk of exposure and infection, rather than individual-level factors such as health behaviors and comorbidities.

**Conclusions:**

Policy- and systems-level interventions should acknowledge and address these social determinants of heightened risk, and future research should evaluate the effects of such interventions to avoid further exacerbation of health inequities during the current and future pandemics.

## Introduction

Infectious disease outbreaks such as the coronavirus disease 2019 (COVID-19) that necessitate drastic public health measures (e.g., social distancing, school, business, and facility closures) have the potential to differentially impact disadvantaged populations and contribute to higher burdens of morbidity and mortality among certain groups. In the United States, the ongoing effects of COVID-19 have varied by region and community over time, and while the full extent of its effect on disadvantaged communities remains uncertain, health disparities are already apparent.

A recent systematic review found that African American (AA)/black and Latino adults experienced disproportionately higher rates of both severe acute respiratory syndrome coronavirus 2 (SARS-CoV-2) infections and COVID-19-related mortality. The authors suggest that differences in exposure and access to care might be driving the higher infection and mortality rates.^1^

Similar disparities in health outcomes have been observed in past infectious disease outbreaks. In the 2009 H1N1 pandemic, AA/blacks and Latinos had higher rates of hospitalizations and mortality in Illinois.^2^ Patients of low socioeconomic status (SES) were also found to have higher odds of hospitalization in New York City.^3^ While it remains too early to know the true impact of SARS-CoV-2 on vulnerable populations, it is likely the United States will feel the ripple effects of these unequal health outcomes for years. For this reason, research on the root causes of these disparities is crucial.

## Methods

This is part of a larger systematic review commissioned by the Veterans Health Administration (VHA) that examined the mediating factors contributing to health-related inequalities during the U.S. epidemics and pandemics predating COVID-19, and the interventions developed to address them.

The protocol, which follows PRISMA guidelines,^4^ was registered to PROSPERO (CRD42020187078) before study initiation.

### Data sources and searches

We searched MEDLINE ALL, PsycINFO, Cochrane Database of Systematic Reviews, and Cochrane Central Register of Controlled Trials from database inception through May 20, 2020. Searches included MeSH terms and free-text words related to previous epidemics, pandemics, disasters, and health disparities. We reviewed the bibliographies of relevant articles and contacted experts to identify additional studies. Search strategies were developed in consultation with a research librarian (Supplementary Appendix SA1).

We further refined search results by performing keyword searches in EndNote (X9.3.3) to exclude articles that were not studies (i.e., errata, comments, replies, and proposals), and studies that were basic science, of animals, not of infectious disease pandemics or epidemics relevant to the United States, and of non-U.S. state or territory populations. Titles and abstracts excluded via keyword search were confirmed by an investigator.

### Study selection

Eligible studies examined mediators of health inequalities by race/ethnicity, SES, disability, or geographic location in primarily adult U.S. populations during the infectious disease epidemics/pandemics predating COVID-19 (Supplementary Appendix SA2 and SA3). Studies were independently reviewed for inclusion by at least two reviewers. Discordant results were resolved through consensus or a third reviewer.

### Data abstraction and quality assessment

From each article, we abstracted details that related to sample size, setting, population characteristics, and inclusion and exclusion criteria; and findings on factors that may contribute to health inequalities. Data were abstracted by one investigator and confirmed by a second. Two reviewers independently assessed study risk-of-bias using modified versions of the Newcastle/Ottawa Scale for observational studies and the Critical Appraisal Skills Programme (CASP) Qualitative Checklist for qualitative studies^5,6^ (Supplementary Appendix SA4). Disagreements were resolved by consensus or a third reviewer.

### Data synthesis

We qualitatively synthesized the evidence and present it in tables. Our approach was guided by a framework that was developed in 2008 by Blumenshine et al.^7^ to describe the mechanisms through which inequalities in influenza health outcomes occur. It was later adapted by Quinn et al. to describe evidence from the H1N1 pandemic.^8,9^

Factors contributing to inequalities are categorized into those related to the following: (1) exposure (factors that affect exposure to infectious agents, including structural factors such as working and living conditions; work-related factors such as the inability to work from home or fear of job loss; and other factors related to childcare or public transportation), (2) susceptibility (factors that increase susceptibility to being infected or having poor outcomes once infected, including existing chronic conditions), (3) access to care (e.g., lack of a regular health care provider, insurance, or the ability to pay coinsurance or copays), and (4) experiencing health care-related discrimination (e.g., self-reported perceived discrimination by a provider).

We expanded exposure-related factors to also include (1) hygiene and health-related behaviors, and (2) information and knowledge. In addition, we broadened factors related to health care discrimination from the experiences of interpersonal mistreatment to also include community discrimination and trust in health care systems and government.

## Results

**FIG. 1.**
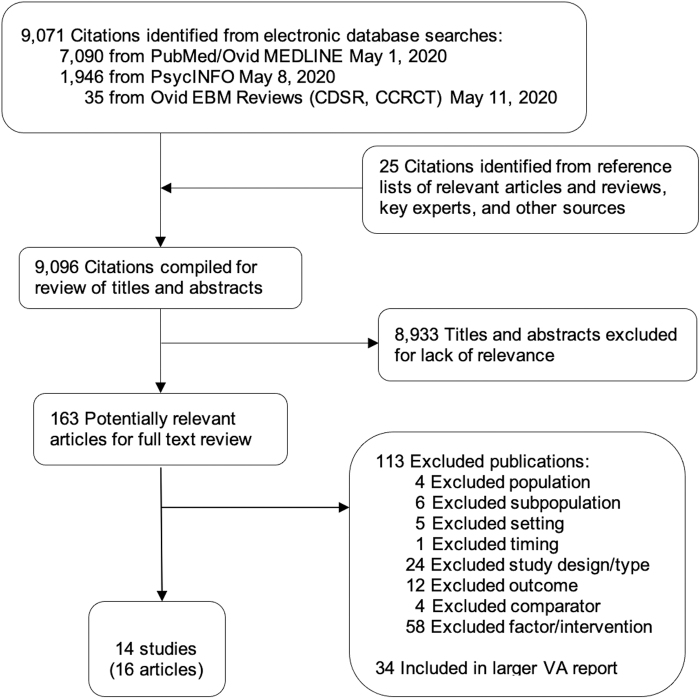
Literature flowchart.

We reviewed 9096 titles and abstracts and 163 full-text articles, and 14 studies (16 articles) met the inclusion criteria (Fig. 1). All but two studies focused on factors that potentially contributed to health disparities during the 2009 H1N1 influenza pandemic. Other studies examined the 2012 Middle East respiratory syndrome (MERS) outbreak^10^ or planning for future infectious disease epidemics.^11^

Six studies examined both the AA/black and Latino populations.^8,10,12–15^ Two studies examined the Asian populations,^14,16^ and two focused on American Indian/Alaska Natives (AI/AN).^14,17^ Five studies examined participants with limited English proficiency (LEP), four of Latino populations,^8,11,18,19^ and one of Chinese immigrants.^20^ Five studies (six articles) examined persons of low SES,^3,10,12,14,21,22^ and one study was of Veterans with spinal cord disorders (Table 1).^12^

**Table 1.** Studies of Risk Factors by Population

Study Author, year ***N*** participants Setting Dates Study design Study timing ***Focus***	Demographics % female Age (SD) Race/ethnicity Education Unemployed ***Other***	AA/black	Latino	Asian and/or Pacific Islander	American Indian or Alaska Native	Limited English proficient	Low socioeconomic status	Rural	Disability	Applicability	Study quality ratings and concerns
Etingen 2012^12^ *N*=3384 Hines, Illinois Veterans with Spinal Cord Disabilities August 2010 Cross-sectional Survey *H1N1*	3% Female Age: 61.82 (11.70) AA/black: 14.71% Latino: 8.42% White: 73.05% <HS: 6.39% HS: 20.21%	✓	✓				✓		✓	Fair	Veteran sample, low response rate
Freimuth, 2014^15^ *N*=1543 Nationally Represented June–July 2009 Cross-sectional Survey *H1N1*	51.8% Female Age: 46.3 (0.54) AA/black: 11.4% Latino:13.7% White: 68.8% <HS: 13.6% HS: 31.7%	✓	✓							Good	—
Hennessy, 2015^17^ *N*=381 AI/AN *N*=72 AK, AZ, NM, OK, and WY 2009 Case Control *H1N1 Mortality*	Cases/controls Female: 50% vs. 54% ≤20: 19% vs. 50%; 21–60: 59% vs. 47%; ≥61: 22% vs. 3% AI/AN: 17% vs. 11% AA/black: 4% vs. 1% Asian: 0% vs. 6% White: 76% vs. 72%				✓					Fair	Issues with method of ascertainment and comparability of cases and controls.
Kumar, 2012^13^ Kumar, 2012^25^ *N*=2042 Nationally Representative January 2010 Cross-sectional Survey *H1N1*	Women: 51.7% Age: 44.9 (SE=0.4) AA/black: 28.94% Latino: 29.48% White: 41.58% <$25K: 30.5% < HS: 18.8%	✓	✓				✓			Good	—
Levy, 2013^3^ *N*=374 New York City October 2009–February 2010 Case Control *H1N1 Hospitalization*	Cases/controls 58% vs. 57% Female Age: 47 vs. 42 AA/black: 22% vs. 9% Latino: 24% vs. 12% White: 22% vs. 46% <HS: 35% vs. 5%; HS: 41% vs. 44%						✓			Fair	Expected uneven response rate, so controls were oversampled. Matched 2:1 as planned.
Lin, 2014^21^ Lin, 2018^22^ *N*=1569 Nationally Representative February–March 2010 Cross-sectional Survey *H1N1*	51% Female Age: 49% were ≤44 AA/black: 11% Latino: 14% White: 68% <HS 14% Unemployed: 21%						✓			Good	—
Lin, 2017^10^ *N*=627 Nationally Representative December 2013 Cross-sectional Survey *MERS and Previous Pandemics*	51% Female 18–29: 19%; 30–39: 15%; 40–49: 20%; 50–59: 20%; ≥60: 26% AA/black 13% Latino 17% White 69% <HS 12%	✓	✓				✓			Fair	Unclear whether confounding factors were controlled.
McCauley, 2013^19^ *N*=46 Four Communities in New England Focus Groups *H1N1*	City A Only: 57.7% Female Age: 45 AA/black: 81.25% Latino: 12.5% White: 6.25% <HS: 43.75%; HS: 43.75% <Poverty: 50%	✓								Poor	Qualitative synthesis not very more robust.
Mesch, 2015^24^ *N*=968 National October 2009 Cross-sectional Survey *H1N1*	51% Female Age: 45.80 (17.84) AA/black: 11% Latino: 7.6% <HS: 14.2% HS: 30.9%	✓	✓				✓			Good	Unclear if respondents similar to nonrespondents.
Quinn, 2009^23^ Quinn, 2011^8^ 2009 *N*=1543 2011 *N*=1479 Nationally Representative June 2009 Cross-sectional Study *H1N1*	2009 51.8% Female Age: 46.3 AA/black: 11.4% Latino: 13.7% <HS: 13.6%; HS: 31.7% 2011 51.2% Female 18–34: 27.9%; 35–64: 57%; ≥65: 15.1% AA/black: 13.1% Latino (LEP): 15.5% English-speaking Latino: 4.4% <$25K: 25.5% <HS: 14%	✓	✓			✓				Good	—
Schoch-Spana, 2010^18^ *N*=33 Multiple sites nationally July–October 2009 Qualitative stakeholder interviews *H1N1*	18 Community clinic executives 6 Government agencies 7 MSFW advocacy groups 2 Industry and academic contacts		✓			✓				Good	Stakeholder interviews, qualitative methods not adequately described.
SteelFisher, 2015^14^ *N*=2355 Nationally Representative March–April 2010 Cross-sectional Survey *H1N1*	Female: NR AA/black: 14.3% Latino: 13.8% Asian: 11.7% AI/AN: 11.4% White: 48.8%	✓	✓	✓	✓		✓			Good	—
Witrago, 2011^11^ *N*=209 Fresno County, CA Cross-sectional Survey *Influenza Pandemic Preparedness: Rural Latinos*	71% Female 18–34: 35%; 35–54: 57%; 55–64: 5%; 65 older: 1% Born in Mexico: 89% <HS: 53%		✓			✓		✓		Fair	No control for confounders, methods poorly reported.
Yip, 2009^20^ *N*=100 Seattle, WA April–June, 2009 Cross-sectional Survey *H1N1*	73% Female Age: Median 47.5 Years in the U.S.: 0–5: 39%; 6–10: 27%; >10: 35%					✓				Fair	Pilot study, no control for confounders, methods poorly reported.

AA, African American; AI/AN, American Indian/Alaska Native; HS, high school; I, intervention; LEP, limited English proficiency; MSFW, migrant and seasonal farmworkers; SD, standard deviation; SES, socioeconomic status; U.S., United States.

Within each subsection, we organized findings in the following order: (1) associations between demographic characteristics (e.g., race/ethnicity and SES) and hypothesized mediating risk factors (e.g., exposure and susceptibility), (2) associations between risk factors and outcomes (e.g., influenza-like illness and hospitalization), (3) evidence of mediation of associations between demographic characteristics and outcomes by hypothesized risk factors, and (4) findings from qualitative studies. Supplementary Appendix SA5 provides the study detail.

### Exposure

Five studies (six articles) examined the factors that related to exposure.^8,13,14,18,21,22^ Table 1 provides study population detail. Table 2 highlights study findings by population by exposure subcategory.

**Table 2.** Health Inequity Risk Factors by Group: Exposure

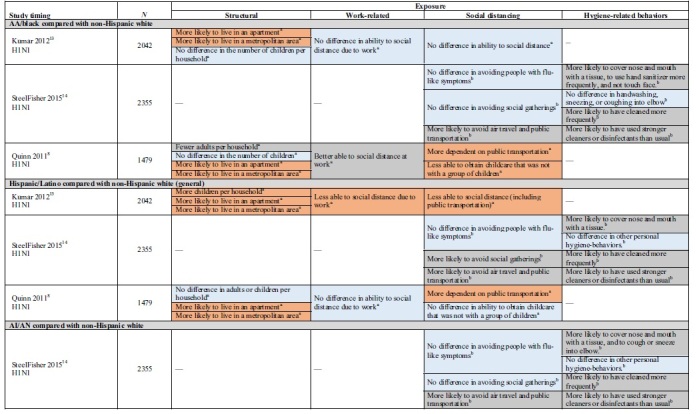 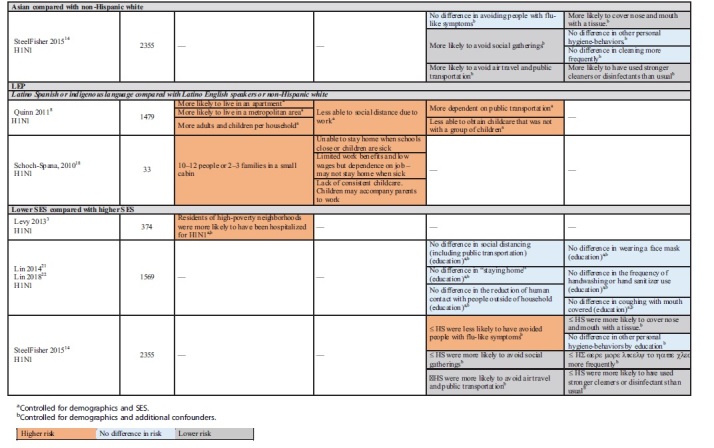

#### Structural

Three studies examined structural factors of exposure.^8,13,18^ Two nationally representative cross-sectional studies examined measures of residential density by race and ethnicity. After controlling for sociodemographics, the studies by Kumar et al. (*N*=2042)^13^ and Quinn et al. (*N*=1479)^8^ both found that, compared with whites, AA/black and Latino (both English-speaking and with LEP) participants were more likely to live in apartments and metropolitan areas.

Both studies also found that AA/black participants had a similar number of or fewer children per household than whites, and Kumar et al. found that Latino families reported significantly more children per household than whites.^13^ Quinn et al. disaggregated Latinos by language and found that while English-speaking Latino participants reported fewer children per household, Latino participants with LEP reported more.^8^

Kumar et al. also examined the relationship between structural factors and the likelihood of a self-reported influenza-like illness (ILI) in both the participants and their household. They found that the number of children in a household was positively related to the likelihood of ILI in the household (odds ratio [OR]=1.10, confidence interval [95% CI] NR). Neither apartment nor metropolitan living was related.^13^

No studies directly addressed the role of structural factors of exposure in mediating disparities in epidemic-related outcomes.

Schoch-Spana et al. interviewed stakeholders from government agencies, community health clinics, advocacy groups, and academia engaged in work related to H1N1 and Latino migrant and seasonal farmworkers (*N*=33). Study methods were not well described. Stakeholders acknowledged that labor camp living conditions, with 10–12 people or 2–3 families sharing small cabins, inhibited compliance with official H1N1 containment guidance (Tables 1 and 2).^18^

#### Work related

The same three studies also looked at work-related exposure factors. The studies by both Quinn et al.^8^ and by Kumar et al.^13^ used a work-related social distancing index that assessed the ease or difficulty of staying home from work if needed (e.g., paid sick leave and the ability to work from home), and found that compared with whites, AA/black participants were similarly^13^ or better able to social distance.^8^ Kumar et al. found that Latinos were less able to social distance.^13^ Examining the role of LEP, Quinn et al. found that there was no difference between English-speaking Latinos and whites, but that Latinos with LEP reported more work-related barriers to social distancing.^8^

Kumar et al.^13^ also examined the relationship between work-related factors and self-reported ILI and found an 8% increase in the likelihood of ILI in the participant and a 6% increase in the household for each unit increase in the social distancing index.

No studies directly addressed the role of work-related exposure factors in mediating disparities in epidemic-related outcomes.

Findings from Schoch-Spana et al.'s qualitative study suggest that Latino migrant and seasonal farmworkers have numerous barriers to staying home from work. As a result, it is not uncommon for them to go to work when sick and also to take their (sick or well) children with them (Tables 1 and 2).^18^

#### Social distancing

Four studies (five articles) examined measures of social distancing by race, ethnicity, and SES.^8,13,14,21,22^ Across studies, findings comparing AA/black and Latino with white participants were inconsistent. Two studies found that AA/black participants were similar to whites or were more likely to social distance (e.g., avoid public transportation, air travel, social gatherings, and people with flu-like symptoms).^13,14^ However, Quinn et al. found that AA/blacks reported being more dependent on public transportation.^8^ One study found that Latinos were better able to social distance.^14^ However, both Quinn et al.^8^ and Kumar et al.^13^ found that both English-speaking Latinos and those with LEP reported more barriers to social distancing than whites.

Quinn et al. found that AA/black, and Latino participants with LEP, reported more difficulty than whites in securing childcare that was not with a group of children. However, English-speaking Latinos and whites were similar.^8^

Only one study (*N*=2355) examined the Asian and AI/AN populations, and it found that both groups were as or more likely than whites to avoid social gatherings, air travel, and public transportation, and to avoid people with flu-like symptoms during the H1N1 pandemic.^14^

Two studies (three articles) examining the relationship between SES and social distancing also reported conflicting findings.^14,21,22^ One was a cross-sectional study (reported in two articles) of the H1N1 pandemic (*N*=1569) that found no association between educational attainment and “staying home,” social distancing (including using public transportation), or the reduction of contact with nonhousehold members.^21,22^ The other found that participants with less than a high school education were more likely to avoid social gatherings, air travel, and public transportation, but were less likely to avoid people with flu-like symptoms.^14^

Kumar et al. examined the relationship between dependence on public transportation and self-reported ILI in the self and household. No differences were reported (Tables 1 and 2).^13^

No studies directly addressed the role of social distancing in mediating disparities in epidemic-related outcomes.

#### Hygiene-related behaviors

Two cross-sectional studies (three articles) examined hygiene-related behaviors during the 2009 H1N1 pandemic.^14,21,22^ Findings were consistent that participants with lower education attainment were as or more likely than those with some college or college graduates to report adhering to recommended cleaning and hygiene practices during H1N1 (e.g., frequent handwashing, hand sanitizer, coughing with mouth covered, and cleaning more frequently). One study compared white with AA/black, Latino, Asian, and AI/AN participants. Racial and ethnic minority participants were no less likely to report following recommended cleaning and personal hygiene practices (Tables 1 and 2).^14^

No studies examined the associations of hygiene-related behaviors with epidemic-related outcomes, or the role of hygiene-related behaviors as mediators of racial or socioeconomic disparities in outcomes.

### Susceptibility

One cross-sectional study examined comorbid conditions associated with susceptibility to H1N1 complications and found that AA/black, English-speaking Latino participants, and Latino participants with LEP all had similar or fewer comorbid conditions than whites.^8^

Two case/control studies examined the relationship between comorbid conditions associated with susceptibility to H1N1 complications, and patient outcomes. One study (*N*=374) was conducted in New York City, and looked at the impact of both education and neighborhood poverty on hospitalization for H1N1. It found that overall, adults with one or more comorbid conditions associated with susceptibility to H1N1 complications were significantly more likely to be hospitalized (OR=12.83, 95% CI [4.99–32.97]).

In a model adjusted for education and access to care, adults with one or more comorbid conditions remained more likely to be hospitalized (adjusted odds ratio [AOR]=7.61, 95% CI [2.68–21.65]). Comorbid conditions (and access to care) only partially mediated the relationship between education and hospitalization. After adjustment, compared with adults with some college or more, both adults with less than or equal to a high school education (AOR=21.21, 95% CI [5.32–84.53]) and high school graduates (AOR=3.82, 95% CI [1.64–8.90]) were still more likely to be hospitalized.^3^

The findings were similar for the relationship between neighborhood poverty and hospitalization. After controlling for access to care and the percentage of residents below the federal poverty level (FPL) in a neighborhood, adults with one or more comorbid conditions had 10 times higher odds of hospitalization (AOR=10.05, 95% CI [3.65–27.64]). After adjusting for comorbid conditions (and access to care), adults from neighborhoods with 30% or more residents under the FPL remained at five times greater odds of hospitalization (AOR=5.02, 95% CI [1.83–13.89]).^3^

The second case/control study (*N*=381) found that although AI/AN were nearly twice as likely to die from H1N1 (OR=1.95, 95% CI [1.03–3.68]), comorbid conditions and age mediated the relationship, and AI/AN race was not an independent risk factor for H1N1 mortality.^17^

### Access to care

Three cross-sectional studies^8,11,14^ and a qualitative study^18^ examined factors that related to access to care during H1N1. Findings indicated that AA/black and English-speaking Latino participants were no different from whites on an access to care index measure (Table 3). However, Latinos with LEP had significantly lower access.^8^

**Table 3.** Health Inequity Risk Factors by Group: Susceptibility, Access to Care, Discrimination and Trust, Information and Knowledge

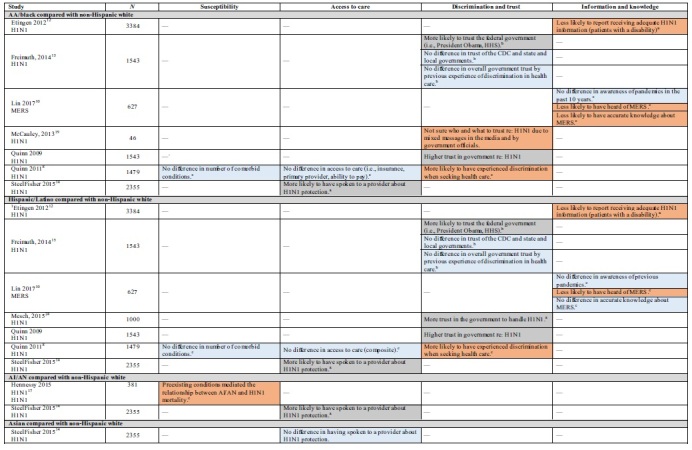 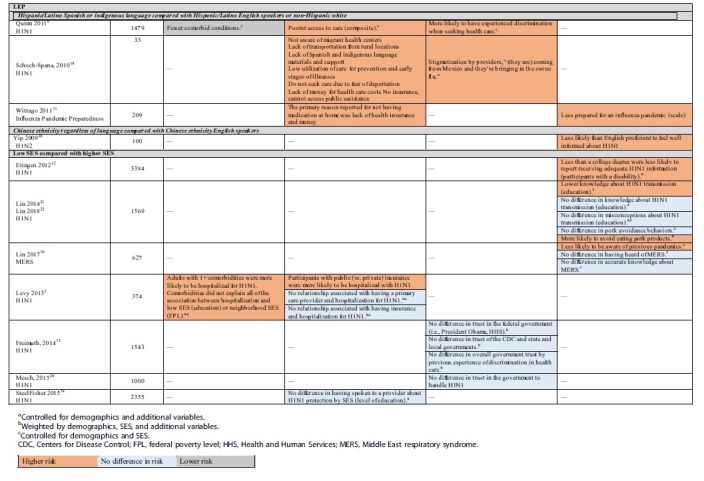

In addition, when controlling for demographics, access to health care, and H1N1-related attitudes, AA/black, Latino, and AI/AN participants were more likely than whites to have spoken to a doctor or other health care professional about how to protect themselves or their families from H1N1. There was no difference for Asians or low SES participants.^14^ Other studies found that among LEP Latinos, the primary reason for not having medication on hand in case of an influenza pandemic was the lack of health insurance (Tables 1 and 3). ^11^

One additional study^3^ examined the relationship between measures of access to care and hospitalization for H1N1 in New York City. There was no difference in hospitalization when comparing adults with and without health insurance (OR=0.42, 95% CI [0.12–1.49]). However, people with private (vs. public) insurance were less likely to be hospitalized (OR=0.15, 95% CI [0.07–0.32]). There was no significant relationship between having a primary care provider and hospitalization for H1N1 (OR=0.88, 95% CI [0.35–2.18]).

In a model adjusted for participant education and comorbid conditions, the relationships between having a primary care provider (AOR=1.88, 95% CI [0.50–7.05]) and having health insurance (AOR=0.73, 95% CI [0.14–3.70]) and hospitalization for H1N1 remained nonsignificant. As previously noted, when adjusting for access to care (and comorbid conditions), compared with adults with some college or more, both adults with less than or equal to a high school education (AOR=21.21, 95% CI [5.32–84.53]) and high school graduates (AOR=3.82, 95% CI [1.64–8.90]) were more likely to be hospitalized.

The findings were similar for the relationship between neighborhood poverty and hospitalization. After controlling for comorbid conditions and the percentage of residents below the FPL in a neighborhood, neither having a primary care provider (AOR=1.50, 95% CI [0.42–5.30]) nor health insurance (AOR=0.42, 95% CI [0.09–2.04]) was significantly associated with hospitalization. As previously noted, after adjusting for access to care (and comorbid conditions), adults from neighborhoods with 30% or more residents under the FPL had 5 times greater odds of hospitalization (AOR=5.02, 95% CI [1.83–13.89]).^3^

A qualitative study of migrant and seasonal farmworkers found that ingrained barriers for low health care utilization such as lack of money for care, lack of insurance or ability to access public assistance, lack of knowledge of migrant health clinics, lack of Spanish- and indigenous language materials and support at health centers, lack of transportation, and fear of deportation would likely impede treatment for H1N1.^18^

### Discrimination and trust

One cross-sectional study^8^ and one qualitative study^18^ of H1N1 pandemic-related disparities examined experiences of discrimination in health care settings. Findings indicated that compared with whites, AA/black and Latino participants (both English-speaking and LEP) were more likely to have ever experienced discrimination when seeking health care.^8^

Three cross-sectional studies^15,23,24^ and one qualitative study^19^ examined trust in the government and government agencies regarding the handling of H1N1. Findings indicated similar or higher government trust scores among AA/black and Latino participants.^23,24^ AA/black and Latino participants were more likely than whites to trust the federal government, including President Obama specifically, with no difference for the Centers for Disease Control (CDC), or state or local governments.^15^ There was no difference in trust by SES (Tables 1 and 3).^15^

No studies examined associations of discrimination or trust with epidemic-related outcomes, or the role of discrimination and trust as mediators of racial or socioeconomic disparities in outcomes.

Findings from a qualitative study suggest that AA/black participants were unsure whom to trust with regard to H1N1 due to mixed messages from the media and government officials.^19^ In another, health care providers reported knowledge of stigmatization directed toward Latino migrant and seasonal farmworkers by other providers. For example, one participant reported overhearing a colleague say, “People [are] coming from Mexico and they're bringing in the swine flu” (Table 1 and 3).^18^

### Information and knowledge

Five cross-sectional studies examined factors related to information and knowledge of pandemics predating COVID-19. A study of Veterans with spinal cord injuries (*N*=3384) found that, compared to whites, fewer AA/black and Latino Veterans, and fewer low SES participants reported receiving “adequate” information about H1N1.^12^

Another study asked respondents about the MERS outbreak and previous pandemics. AA/black and Latino participants were similar to whites in their awareness of previous pandemics; however, participants with lower SES (education) had less awareness. Both AA/blacks and Latino participants were less likely than whites to have heard of MERS, and AA/black participants were less likely to have accurate knowledge about MERS. There was no difference by SES for having heard of, or having accurate knowledge of, MERS (Tables 1 and 3).^10^

Two studies examined information and knowledge by English proficiency. A small cross-sectional survey of Latinos in a rural setting in California (*N*=209) compared English-speaking Latinos with LEP Latinos and found that those with LEP scored lower on an influenza pandemic preparedness scale.^11^ The second was a study of Chinese residents in Seattle. It found that compared with those with better English skills, LEP participants were less likely to feel well-informed about H1N1. Commonly used channels for information among LEP participants were the television (including Chinese-language channels; 81%), Chinese-language newspapers (69%), and community-based organizations (30%; Tables 1 and 3). The study did not control for confounding variables.^20^

A study (*N*=1569) examined H1N1 knowledge and misconceptions by SES (education) and found that after controlling for sociodemographics and communication behaviors, there were no differences on an H1N1 transmission knowledge and misconception index. However, even after controlling for confounders, participants with less than a high school education were more likely than those with a college degree to avoid eating pork products during the swine flu pandemic.^21,22^

No studies examined associations of information and knowledge with epidemic-related outcomes, or the role of information and knowledge as mediators of racial or socioeconomic disparities in outcomes.

## Discussion

To our knowledge, this is the first review of studies aimed at identifying the factors that mediate health disparities in infectious disease epidemics. Our conceptual framework was guided by the work of Quinn and Kumar, who considered the potential causes of epidemic influenza based on measures of exposure, susceptibility, and access to care as they applied to data collected in 2009–2010 during the H1N1 pandemic. The framework points to proximal and distal determinants of disease burden with the goal of identifying potential points of policy and programmatic intervention.

Our review revealed that disparities during the H1N1 pandemic were more related to differential exposure to the virus than to susceptibility or access to care. Disparities in exposure were, in turn, related to societal structural and work-related factors, rather than individual factors such as hygiene and cleaning. These findings were consistent across studies. We identified few significant differences in social distancing attitudes and intentions between groups. Instead, it was clear that the meaningful differences lay in the ability or inability to social distance.

Only one study examining variables related to exposure to illness disaggregated the Latino population by language proficiency, and one additional study provided qualitative input in the form of stakeholder interviews. In contrast with other populations we examined, compared with either English-proficient Latinos or whites, limited English-proficient Latinos (and/or migrant and seasonal farmworkers) were at higher risk across both structural and work-related variables measured (Table 2).

Susceptibility to illness played a major role in H1N1 severity and mortality, although disparities in H1N1 outcomes did not appear to be attributable to differential comorbidity burden across socioeconomic groups. Access to care (i.e., having a primary care provider or health insurance) also did not appear to play a major role in explaining disparities in H1N1 outcomes.

Significantly greater proportions of every racial and ethnic minority group reported having experienced discrimination while seeking health care, and many reported being less informed or were less prepared. Much of the literature guiding communication is dated due to advances in technology, and findings of proportionally higher rates of trust in the federal government, particularly in AA/black and Latino adults, may be out of date (Table 3).

Many of the studies are a decade old, most examined the H1N1 pandemic, and findings may no longer be applicable. Much has changed over the last decade, including advances in technology that affect the ways that we communicate, access information, and interact with health care providers. In recent years, our country has shifted sociopolitically, affecting factors related to discrimination and government trust among different population subgroups. In addition, the COVID-19 pandemic is very different than H1N1. Not only is SARS-CoV-2 more infectious and more widely spread in the United States, but it has affected the way that we live, work, and even socialize in more pervasive ways that the H1N1 pandemic did.

However, despite a decade of change, some things have remained constant. The societal factors that placed vulnerable populations at higher risk for health disparities are largely unchanged. Groups that were vulnerable a decade ago are similarly or more vulnerable today. Social and institutional barriers remain.

As the COVID-19 pandemic enters its third year, it is clear that racial and ethnic minorities are at higher risk than whites.^1^ Policy- and systems-level interventions are urgently needed and should address the social determinants of health and mitigate exposure risk. Potential mitigating strategies include extending eviction moratoriums, financial support for basic needs and health care, and prioritizing those at higher risk due to housing and work-related conditions for both testing and vaccine distribution.

There are a number of limitations of this evidence base. The operationalization of potential mediating factors was heterogeneous, and we identified very few studies that examined whether risk factors mediated the associations between population characteristics and outcomes. Several included studies did not control for confounding variables or ambiguously reported their methods,^10,11,20^ although many studies were well conducted and adequately reported. In addition, qualitative studies did not clearly report their methodology and/or findings.^18,19^

Many of the studies are a decade old, and in light of advances in technology and sociopolitical shifts over the past decade, their findings may be less applicable now than they were then. The categorization of racial and ethnic groups was not consistent across studies (e.g., Latinos as a group or stratified by nativity or language), and very few studies examined disparities among the Asian, Pacific Islander, and AI/AN populations, or among rural residents and adults with disabilities.

## Conclusion

The literature examining health disparities associated with previous infectious disease epidemics may provide some guidance for the current COVID-19 response. Our findings indicate that pandemic-related disparities emanate primarily from inequalities in social conditions that place racial and ethnic minorities and low SES populations at greater risk of exposure and infection, rather than individual-level factors such as health behaviors and comorbidities. Policy- and systems-level interventions should acknowledge and address these social determinants of heightened risk, and future research should evaluate the effects of such interventions to avoid further exacerbation of health inequities during the current and future pandemics.

## Supplementary Material

Supplemental data

Supplemental data

Supplemental data

Supplemental data

Supplemental data

## Disclaimer

The findings and conclusions in this document are those of the author(s) who are responsible for its contents; the findings and conclusions do not necessarily represent the views of the Department of Veterans Affairs or the U.S. Government. Therefore, no statement in this article should be construed as an official position of the Department of Veterans Affairs. This work is part of a larger systematic review commissioned by the Veterans Health Administration (VHA) related to health-related inequities during U.S. epidemics and pandemics predating COVID-19 (https://www.hsrd.research.va.gov/publications/esp/pre-covid-19-inequalities.pdf?).

## Supplementary Material

Supplementary Appendix SA1

Supplementary Appendix SA2

Supplementary Appendix SA3

Supplementary Appendix SA4

Supplementary Appendix SA5

## Abbreviations Used

AA: African American
AI/AN: American Indian/Alaska Native
CASP: Critical Appraisal Skills Programme
CDC: Centers for Disease Control
FPL: federal poverty level
ILI: influenza-like illness
LEP: limited English proficiency
MERS: Middle East respiratory syndrome
SARS-CoV-2: severe acute respiratory syndrome coronavirus 2
SES: socioeconomic status
VHA: Veterans Health Administration
