# Supplementary material for: Factors Associated with Health Inequalities in Infectious Disease Pandemics Predating COVID-19 in the United States: A Systematic Review
Source: Health Equity. 2022 Mar 24;6(1):254–69. doi: 10.1089/heq.2021.0049 (PMC8985532; doi:10.1089/heq.2021.0049)
Supplement: Supplemental data [file Supp_AppS1.docx]

APPENDIX 1. SEARCH STRATEGIES

**Ovid MEDLINE ALL 1946 to May 01, 2020**

Date searched: May 4, 2020

1 Coronavirus Infections/ or COVID-19.rs. (6243)

2 ((("Corona virinae" or "corona virus" or Coronavirinae or coronavirus or COVID or nCoV) adj4 ("19" or "2019" or novel or new)) or (("Corona virinae" or "corona virus" or Coronavirinae or coronavirus or COVID or nCoV) and (wuhan or china or chinese)) or "Corona virinae19" or "Corona virinae2019" or "corona virus19" or "corona virus2019" or Coronavirinae19 or Coronavirinae-19 or Coronavirinae2019 or Coronavirinae-2019 or coronavirus19 or coronavirus-19 or coronavirus2019 or coronavirus-2019 or COVID19 or COVID-19 or COVID2019 or COVID-2019 or nCOV19 or nCOV-19 or nCOV2019 or nCOV-2019 or SARS-CoV-2 or SARS-CoV2 or SARS-CoV or "severe acute respiratory").ti,ab,hw,kw. (16846)

3 or/1-2 (20340)

4 Healthcare Disparities/ or Health Equity/ or Health Status Disparities/ or Culturally Competent Care/ or Social Determinants of Health/ or Sociology, Medical/ (35656)

5 (disadvantaged or discriminat* or disparat* or disparit* or disproportion* or inequal* or inequit* or unequal or underserved or under-served or (cultural* adj3 compet*) or (social* adj3 determin*)).ti,ab,kf. or (difference or different).ti. (679237)

6 Ethnic Groups/ or Minority Groups/ or African Americans/ or Arabs/ or Asian Americans/ or Hispanic Americans/ or Mexican Americans/ or Indigenous Peoples/ or exp Alaska Natives/ (152741)

7 (ethnic* or race* or racial* or minority or minorities or "people of color" or African-American* or Black or Blacks or Hispanic* or Chican* or Latino* or Latina* or Latinx or Mexican-American* or Asian-American* or Chinese-American or Filipino* or Japanese or Korean or Vietnamese or Native American* or Indian or Indians or indigenous).ti,ab,kf. (711222)

8 Socioeconomic Factors/ or Economic Status/ or exp Employment/ or Homeless Persons/ or Medicaid/ or Medically Uninsured/ or exp Medicare/ or Poverty/ or Poverty Areas/ or Public Assistance/ or Public Housing/ or Social Class/ or "Transients and Migrants"/ or Undocumented Immigrants/ or Veterans/ or Working Poor/ (388391)

9 ("blue collar" or impoverish* or homeless* or immigrant* or indigent or low-income or low-wage or lower-income or Medicaid or Medicare or migrant* or poverty or (public adj (assistance or housing)) or social or socio* or SES or undocumented or uninsured or veteran* or (working adj2 (class or poor))).ti,ab,kf. (876545)

10 Disabled Persons/ or Health Services for Persons with Disabilities/ or Persons With Hearing Impairments/ or Visually Impaired Persons/ or Vulnerable Populations/ (56412)

11 (disabilit* or disabled or blind or deaf or handicapped or ((visual* or hearing or physical*) adj impair*) or vulnerab*).ti,ab,kf. (557881)

12 Rural Health/ or Rural Health Services/ or Rural Population/ or Urban Health/ or Urban Health Services/ or Urban Population/ 146477)

13 (inner-city or metropol* or municipal* or neighborhood* or rural-urban or rural or urban or "New York" or "Los Angeles" or Chicago or Houston or Phoenix of Philadelphia or "San Antonio" or "San Diego" or Dallas or "San Jose" or "San Francisco" or Austin or Jacksonville or "Fort Worth" or Columbus or Charlotte or Indianapolis or Seattle or Denver or Washington or Boston or "El Paso" or Detroit or Nashville or Portland or Memphis or "Oklahoma City" or "Las Vegas" or Louisville or Baltimore or Milwaukee or Albuquerque or Tucson or Fresno or Mesa or Sacramento or Atlanta or "Kansas City" or "Colorado Springs" or Miami or Raleigh or Omaha or "Long Beach" or "Virginia Beach" or Oakland or Minneapolis or Tulsa or Arlington or Tampa or "New Orleans").ti,ab,kf. or (city* or cities or county).ti,kf. (530985)

14 or/4-13 (3076764)

15 3 and 14 (1963)

16 15 not (Beijing or "Hong Kong" or Huangshi or Hubei or Jiangsu or London or Paris or Qingdao or Shanghai or Shenzhen or Tianjin or "Wuhan city" or Zhuhai or Caribbean or Europe* or "South America" or "South Asia" or "Southeast Asia" or "East Asia" or "North Africa" or "East Africa" or "West Africa" or "Southern Africa" or Afghanistan or Albania* or Algeria* or Andorra or Angola or Antigua or Argentin* or Armenia* or Australia* or Austria* or Azerbaijan or Bahamas or Bahrain or Bangladesh* or Barbados or Belarus or Belgium or Belize or Benin or Bhutan* or Bolivia* or Bosnia* or Botswana or Brazil* or Britain* or Brunei or Bulgaria* or "Burkina Faso" or Burundi or "Cabo Verde" or Cambodia* or Cameroon* or Canada or Canadian* or "Central African Republic" or Chad or Chile* or China or Colombia* or Comoros or Congo* or "Costa Rica" or "Cote d'Ivoire" or Croatia* or Cuba or Cyprus or Czechia or Denmark or Djibouti or Dominica or "Dominican Republic" or Ecuador or Egypt* or "El Salvador" or England or "Equatorial Guinea" or Eritrea* or Estonia* or Eswatini or Ethiopia* or Fiji* or Finland or France or French or Gabon or Gambia* or Germany or German* or Ghana or Greece or Grenada or Guatemala* or Guinea or Guinea-Bissau or Guyana or Haiti* or Honduras or Hungary or Iceland or India or Indonesia* or Iran* or Iraq* or Ireland or Israel* or Italy or Jamaica* or Japan or Jordan* or Kazakhstan or Kenya* or Kiribati or Korea or Kosovo or Kuwait or Kyrgyzstan or Laos or Latvia* or Lebanon or Lesotho or Liberia* or Libya* or Liechtenstein or Lithuania* or Luxembourg or Madagascar or Malawi or Malaysia* or Maldives or Mali or Malta or "Marshall Islands" or Mauritania* or Mauritius or Mexico or Micronesia* or Moldova or Monaco or Mongolia* or Montenegro or Morocco or Mozambique or Myanmar or Namibia* or Nauru or Nepal* or Netherlands or "New Zealand" or Nicaragua* or Niger or Nigeria* or Macedonia* or Norway or Oman or Pakistan or Palau or Palestine or Panama or "Papua New Guinea" or Paraguay or Peru or Philippines or Poland or Portugal or Qatar or Romania* or Russia* or Rwanda* or "Saint Kitts" or "Saint Lucia" or "Saint Vincent" or "San Marino" or "Sao Tome" or Saudi* or Scotland or Senegal* or Serbia* or Seychelles or "Sierra Leone" or Singapore* or Slovakia* or Slovenia* or "Solomon Islands" or Somalia* or "South Africa" or "South Sudan" or Spain or "Sri Lanka" or Sudan* or Suriname or Sweden or Switzerland or Syria* or Taiwan* or Tajikistan or Tanzania* or Thailand or "Timor-Leste" or "East Timor" or Togo or Tonga* or Trinidad or Tunisia* or Turkey or Turkmenistan or Tuvalu or Uganda* or Ukraine or "United Arab Emirates" or "United Kingdom" or UK or Uruguay or Uzbekistan or Vanuatu or Venezuela* or Vietnam or Yemen or Zambia* or Zimbabwe*).ti. (1391)

17 limit 16 to english language (1295)

18 limit 17 to yr="2020 -Current" (637)

**Ovid PsycINFO 1806 to May Week 1 2020**

Date searched: May 8, 2020

1 *Pandemics/ or *Epidemics/ or *Disasters/ or *Natural Disasters/ or Emergency Preparedness/ (10503)

2 (disaster* or earthquake* or epidemic* or hurricane* or pandemic* or postdisaster or post-disaster or (public adj3 emergenc*) or H1N1 or SARS or Zika or "severe acute respiratory").ti. (9540)

3 or/1-2 (13615)

4 *Health Disparities/ (6152)

5 (difference* or disadvantaged or discriminat* or disparat* or disparit* or disproportion* or inequal* or inequit* or unequal or underserved or under-served or (cultural* adj3 compet*) or (social* adj3 determin*)).ti. (116879)

6 *"Racial and Ethnic Differences"/ or *"Race and Ethnic Discrimination"/ or *Ethnic Groups/ or *Minority Groups/ or *Alaska Natives/ or *American Indians/ or *Arabs/ or exp *Asians/ or *Blacks/ or *Hawaii Natives/ or *Jews/ or exp *"Latinos/Latinos"/ or *Pacific Islanders/ (92858)

7 (ethnic* or race* or racial* or minority or minorities or "people of color" or African-American* or Black or Blacks or Hispanic* or Chican* or Latino* or Latina* or Latinx or Mexican-American* or Asian-American* or Chinese-American or Filipino* or Japanese or Korean or Vietnamese or Native American* or Indian or Indians or indigenous).ti. (130827)

8 *Disadvantaged/ or *Socioeconomic Status/ or *Lower Class/ or *Lower Income Level/ or *Poverty/ or *Social Class/ or exp *Homeless/ or *Shelters/ or *Social Services/ or *"Uninsured (Health Insurance)"/ or *"Welfare Services (Government)"/ or *Immigration/ or *Migrant Farm Workers/ or *Refugees/ (78171)

9 ("blue collar" or impoverish* or homeless* or immigrant* or indigent or low-income or low-wage or lower-income or Medicaid or Medicare or migrant* or immigrant* or poverty or "public assistance" or "public housing" or socio* or SES or undocumented or uninsured or veteran* or "working class" or "working poor").ti. (75944)

10 exp *Disabilities/ or *Disability Discrimination/ or *"At Risk Populations"/ or *Blind/ or *Deaf/ or *Partially Hearing Impaired/ (84942)

11 (disabilit* or disabled or blind or deaf or handicapped or ((visual* or hearing or physical*) adj impair*) or vulnerab*).ti. (88840)

12 exp *Neighborhoods/ or *Rural Environments/ or *Urban Environments/ (28918)

13 (city or cities or county or inner-city or metropol* or municipal* or neighborhood* or rural-urban or rural or urban or "New York" or "Los Angeles" or Chicago or Houston or Phoenix of Philadelphia or "San Antonio" or "San Diego" or Dallas or "San Jose" or "San Francisco" or Austin or Jacksonville or "Fort Worth" or Columbus or (Charlotte and (SC or Carolina*)) or Indianapolis or Seattle or Denver or Washington or Boston or "El Paso" or Detroit or Nashville or Portland or Memphis or "Oklahoma City" or "Las Vegas" or Louisville or Baltimore or Milwaukee or Albuquerque or Tucson or Fresno or Mesa or Sacramento or Atlanta or "Kansas City" or "Colorado Springs" or Miami or Raleigh or Omaha or "Long Beach" or "Virginia Beach" or Oakland or Minneapolis or Tulsa or Arlington or Tampa or "New Orleans").ti. (59491)

14 or/4-13 (543361)

15 3 and 14 (1823)

16 15 not (Beijing or "Hong Kong" or Huangshi or Hubei or Jiangsu or London or Paris or Qingdao or Shanghai or Shenzhen or Tianjin or "Wuhan city" or Zhuhai or Kathmandu).ti. (1816)

17 limit 16 to english language (1751)

18 17 not (epidemic* adj2 (cigarette or cocaine or crack or diabetes or heroin or obesity or opioid or opioids or smoking or tobacco)).ti,ab.(1701)

19 18 not (cat or cats or dog or dogs or mice or mouse or rat or rats or rodent).ti.(1700)

20 19 not (Caribbean or Europe or "South America" or "South Asia" or "Southeast Asia" or "East Asia" or "North Africa" or "East Africa" or "West Africa" or "Southern Africa" or Afghanistan or Albania or Algeria or Andorra or Angola or Antigua or Argentina Armenia or Australia or Austria or Azerbaijan or Bahamas or Bahrain or Bangladesh or Barbados or Belarus or Belgium or Belize or Benin or Bhutan or Bolivia or Bosnia or Botswana or Brazil or Brunei or Bulgaria or "Burkina Faso" or Burundi or "Cabo Verde" or Cambodia or Cameroon or Canada or "Central African Republic" or Chad or Chile or China or Colombia or Comoros or Congo or "Costa Rica" or "Cote d'Ivoire" or Croatia or Cuba or Cyprus or Czechia or Denmark or Djibouti or Dominica or "Dominican Republic" or Ecuador or Egypt or "El Salvador" or "Equatorial Guinea" or Eritrea or Estonia or Eswatini or Ethiopia or Fiji or Finland or France or Gabon or Gambia or Georgia or Germany or Ghana or Greece or Grenada or Guatemala or Guinea or Guinea-Bissau or Guyana or Haiti or Honduras or Hungary or Iceland or India or Indonesia or Iran or Iraq or Ireland or Israel or Italy or Jamaica or Japan or Jordan or Kazakhstan or Kenya or Kiribati or Korea or Kosovo or Kuwait or Kyrgyzstan or Laos or Latvia or Lebanon or Lesotho or Liberia or Libya or Liechtenstein or Lithuania or Luxembourg or Madagascar or Malawi or Malaysia or Maldives or Mali or Malta or "Marshall Islands" or Mauritania or Mauritius or Mexico or Micronesia or Moldova or Monaco or Mongolia or Montenegro or Morocco or Mozambique or Myanmar or Namibia or Nauru or Nepal or Netherlands or "New Zealand" or Nicaragua or Niger or Nigeria or Macedonia or Norway or Oman or Pakistan or Palau or Palestine or Panama or "Papua New Guinea" or Paraguay or Peru or Philippines or Poland or Portugal or Qatar or Romania or Russia or Rwanda or "Saint Kitts" or "Saint Lucia" or "Saint Vincent" or "San Marino" or "Sao Tome" or Saudi or Senegal or Serbia or Seychelles or Sierra Leone or Singapore or Slovakia or Slovenia or "Solomon Islands" or Somalia or "South Africa" or "South Sudan" or Spain or "Sri Lanka" or Sudan or Suriname or Sweden or Switzerland or Syria or Taiwan or Tajikistan or Tanzania or Thailand or "Timor-Leste" or "East Timore" or Togo or Tonga or Trinidad or Tunisia or Turkey or Turkmenistan or Tuvalu or Uganda or Ukraine or "United Arab Emirates" or "United Kingdom" or UK or Uruguay or Uzbekistan or Vanuatu or Venezuela or Vietnam or Yemen or Zambia or Zimbabwe).ti,lo. (1165)

**EBM Reviews - Cochrane Central Register of Controlled Trials April 2020**

Date searched: May 11, 2020

1 (disaster* or earthquake* or epidemic* or hurricane* or pandemic* or post-disaster or (public adj3 emergenc*) or H1N1 or SARS or Zika or "severe acute respiratory").ti. (1455)

2 (difference* or disadvantaged or discriminat* or disparat* or disparit* or disproportion* or inequal* or inequit* or unequal or underserved or under-served or (cultural* adj3 compet*) or (social* adj3 determin*)).ti. (8858)

3 (ethnic* or race* or racial* or minority or minorities or "people of color" or African-American* or Black or Blacks or Hispanic* or Chican* or Latino* or Latina* or Latinx or Mexican-American* or Asian-American* or Chinese-American or Filipino* or Japanese or Korean or Vietnamese or Native American* or Indian or Indians or indigenous).ti. (18233)

4 ("blue collar" or impoverish* or homeless* or immigrant* or indigent or low-income or low-wage or lower-income or Medicaid or Medicare or migrant* or poverty or (public adj (assistance or housing)) or social or socio* or SES or undocumented or uninsured or veteran* or (working adj2 (class or poor))).ti. (13475)

5 (disabilit* or disabled or blind or deaf or handicapped or ((visual* or hearing or physical*) adj impair*) or vulnerab*).ti. (87945)

6 (city or cities or county or inner-city or metropol* or municipal* or neighborhood* or rural-urban or rural or urban or "New York" or "Los Angeles" or Chicago or Houston or Phoenix of Philadelphia or "San Antonio" or "San Diego" or Dallas or "San Jose" or "San Francisco" or Austin or Jacksonville or "Fort Worth" or Columbus or (Charlotte and (SC or Carolina*)) or Indianapolis or Seattle or Denver or Washington or Boston or "El Paso" or Detroit or Nashville or Portland or Memphis or "Oklahoma City" or "Las Vegas" or Louisville or Baltimore or Milwaukee or Albuquerque or Tucson or Fresno or Mesa or Sacramento or Atlanta or "Kansas City" or "Colorado Springs" or Miami or Raleigh or Omaha or "Long Beach" or "Virginia Beach" or Oakland or Minneapolis or Tulsa or Arlington or Tampa or "New Orleans").ti. (8380)

7 or/2-6 (132248)

8 and/1,7 (112)

9 8 not (Beijing or "Hong Kong" or Huangshi or Hubei or Jiangsu or London or Paris or Qingdao or Shanghai or Shenzhen or Tianjin or "Wuhan city" or Zhuhai or Australia* or Bangladesh or Britain or Canada or China or Europe* or England or France or India or Iran or Ireland or Italy or Japan or Korea or Pakistan or Singapore or Scotland or "South Korea" or Thailand or Turkey or "United Kingdom" or UK).ti. (104)

10 9 not (epidemic* adj2 (cigarette or cocaine or crack or diabetes or heroin or obesity or opioid or opioids or smoking or tobacco)).ti,ab. (104)

11 10 not (cat or cats or dog or dogs or mice or mouse or rat or rats or rodent).ti. (104)

12 11 not (Caribbean or Europe or "South America" or "South Asia" or "Southeast Asia" or "East Asia" or "North Africa" or "East Africa" or "West Africa" or "Southern Africa" or Afghanistan or Albania or Algeria or Andorra or Angola or Antigua or Argentina Armenia or Australia or Austria or Azerbaijan or Bahamas or Bahrain or Bangladesh or Barbados or Belarus or Belgium or Belize or Benin or Bhutan or Bolivia or Bosnia or Botswana or Brazil or Brunei or Bulgaria or "Burkina Faso" or Burundi or "Cabo Verde" or Cambodia or Cameroon or Canada or "Central African Republic" or Chad or Chile or China or Colombia or Comoros or Congo or "Costa Rica" or "Cote d'Ivoire" or Croatia or Cuba or Cyprus or Czechia or Denmark or Djibouti or Dominica or "Dominican Republic" or Ecuador or Egypt or "El Salvador" or "Equatorial Guinea" or Eritrea or Estonia or Eswatini or Ethiopia or Fiji or Finland or France or Gabon or Gambia or Georgia or Germany or Ghana or Greece or Grenada or Guatemala or Guinea or Guinea-Bissau or Guyana or Haiti or Honduras or Hungary or Iceland or India or Indonesia or Iran or Iraq or Ireland or Israel or Italy or Jamaica or Japan or Jordan or Kazakhstan or Kenya or Kiribati or Korea or Kosovo or Kuwait or Kyrgyzstan or Laos or Latvia or Lebanon or Lesotho or Liberia or Libya or Liechtenstein or Lithuania or Luxembourg or Madagascar or Malawi or Malaysia or Maldives or Mali or Malta or "Marshall Islands" or Mauritania or Mauritius or Mexico or Micronesia or Moldova or Monaco or Mongolia or Montenegro or Morocco or Mozambique or Myanmar or Namibia or Nauru or Nepal or Netherlands or "New Zealand" or Nicaragua or Niger or Nigeria or Macedonia or Norway or Oman or Pakistan or Palau or Palestine or Panama or "Papua New Guinea" or Paraguay or Peru or Philippines or Poland or Portugal or Qatar or Romania or Russia or Rwanda or "Saint Kitts" or "Saint Lucia" or "Saint Vincent" or "San Marino" or "Sao Tome" or Saudi or Senegal or Serbia or Seychelles or Sierra Leone or Singapore or Slovakia or Slovenia or "Solomon Islands" or Somalia or "South Africa" or "South Sudan" or Spain or "Sri Lanka" or Sudan or Suriname or Sweden or Switzerland or Syria or Taiwan or Tajikistan or Tanzania or Thailand or "Timor-Leste" or "East Timore" or Togo or Tonga or Trinidad or Tunisia or Turkey or Turkmenistan or Tuvalu or Uganda or Ukraine or "United Arab Emirates" or "United Kingdom" or UK or Uruguay or Uzbekistan or Vanuatu or Venezuela or Vietnam or Yemen or Zambia or Zimbabwe).ti. (95)

**EBM Reviews - Cochrane Central Register of Controlled Trials April 2020**

Date searched: May 11, 2020

1 (disaster* or earthquake* or epidemic* or hurricane* or pandemic* or post-disaster or (public adj3 emergenc*) or H1N1 or SARS or Zika or "severe acute respiratory").ti. (1455)

2 (difference* or disadvantaged or discriminat* or disparat* or disparit* or disproportion* or inequal* or inequit* or unequal or underserved or under-served or (cultural* adj3 compet*) or (social* adj3 determin*)).ti. (8858)

3 (ethnic* or race* or racial* or minority or minorities or "people of color" or African-American* or Black or Blacks or Hispanic* or Chican* or Latino* or Latina* or Latinx or Mexican-American* or Asian-American* or Chinese-American or Filipino* or Japanese or Korean or Vietnamese or Native American* or Indian or Indians or indigenous).ti. (18233)

4 ("blue collar" or impoverish* or homeless* or immigrant* or indigent or low-income or low-wage or lower-income or Medicaid or Medicare or migrant* or poverty or (public adj (assistance or housing)) or social or socio* or SES or undocumented or uninsured or veteran* or (working adj2 (class or poor))).ti. (13475)

5 (disabilit* or disabled or blind or deaf or handicapped or ((visual* or hearing or physical*) adj impair*) or PTSD or post-traumatic stress or posttraumatic stress or vulnerab* or ((severe* or serious* or chronic* or persistent*) adj mental* ill*)).ti. (91871)

6 (city or cities or county or inner-city or metropol* or municipal* or neighborhood* or rural-urban or rural or urban or "New York" or "Los Angeles" or Chicago or Houston or Phoenix of Philadelphia or "San Antonio" or "San Diego" or Dallas or "San Jose" or "San Francisco" or Austin or Jacksonville or "Fort Worth" or Columbus or (Charlotte and (SC or Carolina*)) or Indianapolis or Seattle or Denver or Washington or Boston or "El Paso" or Detroit or Nashville or Portland or Memphis or "Oklahoma City" or "Las Vegas" or Louisville or Baltimore or Milwaukee or Albuquerque or Tucson or Fresno or Mesa or Sacramento or Atlanta or "Kansas City" or "Colorado Springs" or Miami or Raleigh or Omaha or "Long Beach" or "Virginia Beach" or Oakland or Minneapolis or Tulsa or Arlington or Tampa or "New Orleans").ti. (8380)

7 or/2-6 (135518)

8 and/1,7 (138)

9 8 not (Beijing or "Hong Kong" or Huangshi or Hubei or Jiangsu or London or Paris or Qingdao or Shanghai or Shenzhen or Tianjin or "Wuhan city" or Zhuhai or Australia* or Bangladesh or Britain or Canada or China or Europe* or England or France or India or Iran or Ireland or Italy or Japan or Korea or Pakistan or Singapore or Scotland or "South Korea" or Thailand or Turkey or "United Kingdom" or UK).ti. (126)

10 9 not (epidemic* adj2 (cigarette or cocaine or crack or diabetes or heroin or obesity or opioid or opioids or smoking or tobacco)).ti,ab. (126)

11 10 not (cat or cats or dog or dogs or mice or mouse or rat or rats or rodent).ti. (126)

12 11 not (Caribbean or Europe or "South America" or "South Asia" or "Southeast Asia" or "East Asia" or "North Africa" or "East Africa" or "West Africa" or "Southern Africa" or Afghanistan or Albania or Algeria or Andorra or Angola or Antigua or Argentina Armenia or Australia or Austria or Azerbaijan or Bahamas or Bahrain or Bangladesh or Barbados or Belarus or Belgium or Belize or Benin or Bhutan or Bolivia or Bosnia or Botswana or Brazil or Brunei or Bulgaria or "Burkina Faso" or Burundi or "Cabo Verde" or Cambodia or Cameroon or Canada or "Central African Republic" or Chad or Chile or China or Colombia or Comoros or Congo or "Costa Rica" or "Cote d'Ivoire" or Croatia or Cuba or Cyprus or Czechia or Denmark or Djibouti or Dominica or "Dominican Republic" or Ecuador or Egypt or "El Salvador" or "Equatorial Guinea" or Eritrea or Estonia or Eswatini or Ethiopia or Fiji or Finland or France or Gabon or Gambia or Georgia or Germany or Ghana or Greece or Grenada or Guatemala or Guinea or Guinea-Bissau or Guyana or Haiti or Honduras or Hungary or Iceland or India or Indonesia or Iran or Iraq or Ireland or Israel or Italy or Jamaica or Japan or Jordan or Kazakhstan or Kenya or Kiribati or Korea or Kosovo or Kuwait or Kyrgyzstan or Laos or Latvia or Lebanon or Lesotho or Liberia or Libya or Liechtenstein or Lithuania or Luxembourg or Madagascar or Malawi or Malaysia or Maldives or Mali or Malta or "Marshall Islands" or Mauritania or Mauritius or Mexico or Micronesia or Moldova or Monaco or Mongolia or Montenegro or Morocco or Mozambique or Myanmar or Namibia or Nauru or Nepal or Netherlands or "New Zealand" or Nicaragua or Niger or Nigeria or Macedonia or Norway or Oman or Pakistan or Palau or Palestine or Panama or "Papua New Guinea" or Paraguay or Peru or Philippines or Poland or Portugal or Qatar or Romania or Russia or Rwanda or "Saint Kitts" or "Saint Lucia" or "Saint Vincent" or "San Marino" or "Sao Tome" or Saudi or Senegal or Serbia or Seychelles or Sierra Leone or Singapore or Slovakia or Slovenia or "Solomon Islands" or Somalia or "South Africa" or "South Sudan" or Spain or "Sri Lanka" or Sudan or Suriname or Sweden or Switzerland or Syria or Taiwan or Tajikistan or Tanzania or Thailand or "Timor-Leste" or "East Timore" or Togo or Tonga or Trinidad or Tunisia or Turkey or Turkmenistan or Tuvalu or Uganda or Ukraine or "United Arab Emirates" or "United Kingdom" or UK or Uruguay or Uzbekistan or Vanuatu or Venezuela or Vietnam or Yemen or Zambia or Zimbabwe).ti. (117)
