# Supplementary material for: Factors Associated with Health Inequalities in Infectious Disease Pandemics Predating COVID-19 in the United States: A Systematic Review
Source: Health Equity. 2022 Mar 24;6(1):254–69. doi: 10.1089/heq.2021.0049 (PMC8985532; doi:10.1089/heq.2021.0049)
Supplement: Supplemental data [file Supp_AppS2.docx]

APPENDIX 2. STUDY SELECTION CRITERIA

1. **Language**: Is the full text of the article in English?

Yes ⭢ Proceed to #2

No ⭢ Code **X1** (NA Language). STOP

1. **Population**: Are participants Adults?

*In mixed-age studies, findings must be reported separately for adults.*

Yes ⭢ Proceed to #3

No ⭢ **Code** **X2** (NA population). Add code **B** if retaining for background/discussion. STOP

1. **Population**: Does the study provide data specific to or stratified by 1 or more of the following participant populations: racial/ethnic minorities or persons a) with disabilities; b) of low socioeconomic status; c) living in rural communities; d) living in population dense neighborhoods; e) living in high-poverty neighborhoods.

*Note: For SES, proxies may include occupation, education, neighborhoods or geographic regions, income, insurance-status, Medicare/Medicaid, SNAP, TANF, households with children qualifying for free or reduced school lunch,*

Yes ⭢ Proceed to #4

No ⭢ **Code X3** (NA subpopulation)**.** Add code **B** if retaining for background/discussion. STOP

1. **Setting**: Is this a US-only population (including U.S territories)?

Yes ⭢ Proceed to #5

No ⭢ **Code X4** (NA setting). Add code **B** if retaining for background/discussion. STOP

1. **Timing**: Is the study performed during or in preparation or response to an infectious disease pandemic or epidemic, or a disaster?

Yes ⭢ Proceed to #6

No ⭢ Code **X5** (NA timing)**.** Add code **B** if retaining for background/discussion. STOP

1. **Study design and type**: Is the article an original research study, or a systematic review/meta-analysis?

*Excluded: Dissertations, non-systematic reviews, conference abstracts, protocols, erratum, comments, non-research letters*

Yes ⭢ Proceed to #7

No ⭢ Code **X6** (NA study design/type)**.** Add code **B** if retaining for background/discussion. STOP

1. **Outcomes**: Is the outcome health-related?

*Examples: Utilization or access, infection, burden of illness, severity, mortality, morbidity*

*Note: Outcomes may also be factors that contribute to disparities in health-related outcome (eg, trust)*

Yes ⭢ Proceed to #9

No ⭢ Proceed to #8

1. **Outcomes**: Is the outcome a result of measures implemented during a pandemic, epidemic, or disaster (*eg,* social distancing, school closure, etc.) that impact a health-related outcome?

*Examples: Employment loss or reduction, income reduction*

Yes ⭢ Proceed to #9

No ⭢ **Code X7** (NA outcome). Add code **B** if retaining for background/discussion. STOP

1. **Comparator**: Does the study include a comparison (within the same population or to a relevant comparator); or, if an intervention study, does it compare results to no intervention, pre-intervention, or another intervention or public health response?

Yes ⭢ Proceed to #11

No ⭢ Proceed to #10

1. **Pre-intervention studies**: Does the study inform a future intervention (*eg,* survey, qualitative study)

*Examples:* *Preferred forms of communication, emergency preparedness, social distancing, access to care*

Yes ⭢ Proceed to #11

No ⭢ **Code X8** (NA comparator). Add code **B** if retaining for background/discussion. STOP

1. **KQ1**: Does the study examine the COVID-19 Pandemic?

Yes ⭢ **Code KQ1.** If the study is a systematic review or meta-analysis, **Code KQ1SR**.

STOP

No ⭢ Proceed to #12

1. **Timing**: Is the study performed during, or in preparation or response to, a disaster?

Yes ⭢ Proceed to #14

No ⭢ Proceed to #13

1. **KQ2** **Contributing Factors:** Does the study examine factors that **contribute to** health inequalities?

*Examples: Risk of exposure, susceptibility/risk of poor outcomes, access to care, trust in healthcare system, discrimination.*

*Note: These are not 1^st^ Generation/Phase I studies that simply find that a disparity exists. These are 2^nd^ Generation/Phase II studies that help us to understand why (see Kilbourne et. al, 2006 – uploaded to Slack).*

Yes ⭢ **Code KQ2.** If the study is a systematic review or meta-analysis, **Code KQ2SR**. Proceed to #14

No ⭢ proceed to #14

1. **KQ3**: Is the study a 1) program evaluation, pre-intervention study (*eg,* survey, qualitative), or an intervention study 2) designed to mitigate health inequalities?

*Select “Yes” only if both 1) and 2) are true*

Yes ⭢ If the study is not also included in KQ2, **Code KQ3.** If the study is a systematic review or meta-analysis, **Code KQ3SR**.

Yes ⭢ If the study is also included in KQ2, **Code KQ2&3.** If the study is a systematic review or meta-analysis, **Code KQ2&3SR**.

No ⭢ **Code X9** (NA factors or intervention)**.** Add code **B** if retaining for background/discussion. STOP

*Note: B codes can be added for any excluded study that we should retain/reference for background or discussion.*

**Key Questions**

KQ1: In the COVID-19 pandemic:

1. What health inequalities have been described?
2. What factors have contributed to health inequalities?
3. What is the effectiveness of interventions used to address health inequalities?

KQ2: What factors contribute to disparate infection rates and health-related outcomes among different segments of the population during infectious disease epidemics or pandemics?

KQ3: What interventions have been used to reduce health inequalities in infectious disease transmission or health outcomes in disasters, epidemics or pandemics?

**Codes Key:**

X1: NA Language

X2: NA Population

X3: NA Subpopulation

X4: NA Setting

X5: NA Timing

X6: NA Study design/type

X7: NA Outcome

X8: NA Comparator

X9: NA factor/intervention
