# Supplementary material for: Factors Associated with Health Inequalities in Infectious Disease Pandemics Predating COVID-19 in the United States: A Systematic Review
Source: Health Equity. 2022 Mar 24;6(1):254–69. doi: 10.1089/heq.2021.0049 (PMC8985532; doi:10.1089/heq.2021.0049)
Supplement: Supplemental data [file Supp_AppS3.docx]

**Appendix 3. PICOTS**

|  | **KQ1: What factors contribute to disparate infection rates and health-related outcomes among different segments of the population during infectious disease epidemics or pandemics?** | **KQ2: What interventions or intervention components have been used to reduce health inequalities (or identified in preliminary studies) in infectious disease transmission or health outcomes in disasters, infectious disease epidemics or pandemics in the United States?** |
| --- | --- | --- |
| **Populations** | Adult Subgroups: race or ethnicity, socioeconomic status, disability, geographic location (*eg*, urban/rural, high density neighborhoods) | |
| **Intervention/**  **Mediating and Moderating Factors** | Risk of exposure:   - Structural (employment, urban/rural, living arrangement, crowding) - Work-related inability to social distance - Other measures of inability to social distance (childcare access, need for public transport, language or cultural barriers) - Access to clean water and sanitation - Hygiene and health-related behaviors   Susceptibility:   - Comorbid chronic diseases - Immunosuppression - Psychologic and nutritional stress   Access to care:   - Regular health care provider - Insurance - Quality of health care   Discrimination and trust   - Interpersonal mistreatment - Community discrimination - Trust in healthcare systems and government   Information/Knowledge | - Emergency preparedness - Messaging and communication - Employment, telework - Childcare - Health care access |
| **Comparator** | - Comparison group within the same group - Comparison to other groups relevant to the population | - Standard public health response - No intervention or pre-intervention - Other interventions - No comparator necessary for pre-intervention studies |
| **Outcomes** | - Mortality - Health care utilization and access - Infectious-disease-related hospitalizations - Burden of illness - Severity of illness - Loss of job due to epidemic/pandemic/disaster | |
| **Timing** | Related to an infectious disease pandemic or epidemic | Related to an infectious disease pandemic or epidemic, or disaster |
| **Setting** | United States and Territories | |
| **Study design** | Trials, quasi-experimental, observational, descriptive, case series (depending on search yield), qualitative. Systematic reviews will be included if they directly address key questions. If not, reference lists will be pearled. | |
