# Supplementary material for: Factors Associated with Health Inequalities in Infectious Disease Pandemics Predating COVID-19 in the United States: A Systematic Review
Source: Health Equity. 2022 Mar 24;6(1):254–69. doi: 10.1089/heq.2021.0049 (PMC8985532; doi:10.1089/heq.2021.0049)
Supplement: Supplemental data [file Supp_AppS4.docx]

Appendix 4. Quality Criteria and Ratings

Table. Quality Ratings for Cross-sectional Studies*

| **Author** | **1** | **2** | **3** | **4** | **5** | **6** | **7** | **Quality concerns** | **Applicability(**[**1**](#_ENREF_1)**)** |
| --- | --- | --- | --- | --- | --- | --- | --- | --- | --- |
| Etingen, 2013([2](#_ENREF_2)) | Y | Y | N | Y | Y, A&B | P | Y | Veteran sample. Low response rate. | Fair |
| Freimuth, 2014([3](#_ENREF_3)) | Y | Y | Y | Y | Y, A&B | P | Y | --- | Good |
| Kumar, 2012([4](#_ENREF_4), [5](#_ENREF_5)) | Y | Y | Y | Y | Y, A&B | P | Y | --- | Good |
| Lin, 2014([6](#_ENREF_6)) and 2018([7](#_ENREF_7)) | Y | Y | Y | Y | Y, A&B | P | Y | --- | Good |
| Lin, 2017([8](#_ENREF_8)) | Y | N | Y | Y | U | P | Y | Unclear whether confounding factors were controlled. | Fair |
| Mesch, 2015([9](#_ENREF_9)) | Y | Y | U | Y | Y, A&B | P | Y | Unclear if respondents similar to non-respondents. | Good |
| Quinn, 2009([10](#_ENREF_10))  Quinn, 2011([11](#_ENREF_11)) | Y | Y | Y | Y | Y, A&B | P | Y | --- | Good |
| SteelFisher, 2015([12](#_ENREF_12)) | Y | Y | Y | Y | Y, A&B | P | Y | --- | Good |
| Witrago, 2011([13](#_ENREF_13)) | N | N | NR | Y | N | P | N | No control for confounders, methods poorly reported. | Fair |
| Yip, 2009([14](#_ENREF_14)) | N | U | U | Y | N | P | N | Pilot study, no control for confounders, methods poorly reported. | Fair |

Abbreviations: N=No; NA=Not applicable; NR=Not reported; P=Partial; U=Unclear; Y=Yes

*Criteria (Adapted Newcastle-Ottawa([15](#_ENREF_15))):

Selection

1. Sample representative?

yes = Truly representative of the average in the target population (all subjects or random sampling); or, somewhat representative of the average in the target population. (non-random sampling)

no = Selected group of users

unclear = No description of the sampling strategy.

1. Sample size justified and satisfactory?

Yes/no

1. Non-respondents comparable to respondents?

yes = Comparability between respondents and non-respondents characteristics is established, and the response rate is satisfactory.

no = The response rate is unsatisfactory, or the comparability between respondents and non-respondents is unsatisfactory.

unclear = No description of the response rate or the characteristics of the responders and the non-responders.

1. Ascertainment of the exposure (risk factor) appropriate?

yes = Adequately described

unclear = Not adequate description of the measurement tool.

Comparability

1. The subjects in different outcome groups are comparable, based on the study design or analysis. Confounding factors are controlled.

yes = specify a, b, or a&b

a) The study controls for the most important factor (age).

b) The study control for any additional factor.

no = no adjustment for potential confounders

Outcome

1. Assessment of the outcome appropriate?

Yes = Independent blind assessment or Record linkage.

Partial = Self report.

unclear = No description.

1. Statistical test described and appropriate (including measurement of the association, including CIs and probability level [p- value])?

Yes/no/unclear

Table. Quality Ratings for Qualitative Studies*

| **Author, Year** | **Section A** | | | | **Section B** | | | **Section C** | **Overall Quality Notes** | **Applicability** |
| --- | --- | --- | --- | --- | --- | --- | --- | --- | --- | --- |
|  | **1** | **2** | **3** | **4** | **5** | **6** | **7** | **How valuable is the research?** |  |  |
| McCauley, 2013([16](#_ENREF_16)) | Y | Y | Y | Y | U | Y | Y | Valuable well conducted study. | Qualitative synthesis could have been more robust. | Poor |
| Schoch-Spana, 2010([17](#_ENREF_17)) | U | Y | U | NR | NR | N | U | Valuable, this work can help guide policy. | Stakeholder interviews, qualitative methods not adequately described. | Good |

Abbreviations: N=No; NR=Not reported; U=Unclear; Y=Yes

*Criteria (CASP([18](#_ENREF_18))):

Section A: Are the results valid?

1. Was the research design appropriate to address the aims of the research?
2. Was the recruitment strategy appropriate to the aims of the research?
3. Was the data collected in a way that addressed the research issue?
4. Has the relationship between researcher and participants been adequately considered?

Section B: What are the results?

1. Have ethical issues been taken into consideration?
2. Was the data analysis sufficiently rigorous?
3. Is there a clear statement of findings?

Section C: Will the results help locally?

Table. Quality Ratings for Case Control Studies*

| **Study** | **1** | **2** | **3** | **4** | **5** | **6** | **7** | **8** | **Overall Quality** | **Applicability(**[**1**](#_ENREF_1)**)** |
| --- | --- | --- | --- | --- | --- | --- | --- | --- | --- | --- |
| Hennessy, 2016([19](#_ENREF_19)) | Y | Y | Y | Y | N | Y | N | NA | Issues with method of ascertainment and comparability of cases and controls. | Fair |
| Levy, 2013([20](#_ENREF_20)) | Y | Y | Y | Y | Y, A&B | Y | Y | U | Expected uneven response rate, so controls were oversampled. Matched 2:1 as planned. | Fair |

Abbreviations: N=No; NA=Not applicable; U=Unclear; Y=Yes

*Criteria (Newcastle-Ottawa([15](#_ENREF_15))):

Selection

1. Is the case definition adequate?

Yes = with independent validation

No = record linkage or based on self-reports

Unclear = no description

1. Representativeness of the cases?

Yes = consecutive or obviously representative series of cases

No= potential for selection biases

Unclear = not stated

1. Selection of Controls appropriate?

Yes = community controls

No = hospital controls

Unclear = no description

1. Definition of Controls?

Yes = no history of disease (endpoint)

No/Unclear

Comparability

1. Comparability of cases and controls on the basis of the design or analysis

Yes = specify a or a&b in response

- 1. study controls for the most important factor.
  2. study controls for any additional factor.

No = neither of the above

Exposure

1. Ascertainment of exposure

Yes = secure record (*eg,* surgical records) or structured interview where blind to case/control status

No= interview not blinded to case/control status or written self-report or medical record only

Unclear = no description

1. Same method of ascertainment for cases and controls? Y/N
2. Non-Response rate adequate

Yes = same rate for both groups

No = non respondents just described or rate different and no designation
