# Supplementary material for: Factors Associated with Health Inequalities in Infectious Disease Pandemics Predating COVID-19 in the United States: A Systematic Review
Source: Health Equity. 2022 Mar 24;6(1):254–69. doi: 10.1089/heq.2021.0049 (PMC8985532; doi:10.1089/heq.2021.0049)
Supplement: Supplemental data [file Supp_AppS5.docx]

Appendix 5. Studies Examining Measures of Exposure, Susceptibility, Access to Care, Trust and Discrimination, and Information and Knowledge

| **Author, Year**  **N Participants**  **Focus**  **Population(s) of Interest** | **Mediating Factors** |
| --- | --- |
| **Exposure** | |
| ***Structural*** | |
| Kumar, 2012([5](#_ENREF_5))  N = 2,042  *H1N1*   - AA/Black - Latino | Children per Household:   - Controlling for age, gender, and SES, Latinos had significantly more children per household (b = 0.43; p < 0.001.). - There was no difference between AA/Blacks and Whites. - Each additional child resulted in a 10% increase in the likelihood of an influenza like illness in the household (b = 0.10; p < 0.05).   Density:   - Controlling for age, gender, and SES, Latinos (b = 0.95; p < 0.01) and AA/Blacks (b = 0.66; p < .05) were more likely to live in an apartment building. - Latinos (b = 1.08; p < 0.001) and AA/Blacks (b = 0.64; p < 0.001) were also more likely to live in a metropolitan area. - There was no relationship between density and likelihood of an influenza like illness. |
| Levy 2013([20](#_ENREF_20))  N = 374  *H1N1 Hospitalization*   - Low SES: Neighborhood Poverty | Hospitalization:   - Multivariate analysis that also included SES (education), having a primary care provider, health insurance, and 1+ comorbid condition found that those living in neighborhoods with 30+% poverty were more likely to be hospitalized (AOR = 5.02 [95% CI 1.82-13.89]) |
| Quinn 2011([11](#_ENREF_11))  N = 1,479  *H1N1*   - AA/Black - Latino (English and LEP) | Number of People in a Household:   - Controlling for age, gender, and SES, Spanish-speaking Latinos had more adults (b = 0.55, p < 0.001) and children (b = 0.33, p < 0.001) per households than Whites. - AA/Blacks had fewer adults per household (b = -0.22, p = 0.01). - There was no difference between English-speaking Latinos and Whites.   Apartment and Metropolitan Living:   - Controlling for age, gender, and SES, AA/Blacks (AOR = 2.4, 95% CI [1.3-4.3]); AOR = 3.1, 95% CI [1.9-5.1]), English-speaking Latinos (AOR = 5.1, 95% CI [1.4-19.7]); AOR = 2.1, 95% CI [1.0-4.4]), and Spanish-speaking Latinos (AOR = 3.9, 95% CI [2.1-7.1]); AOR = 3.2, 95% CI [1.8-5.7]) were more likely to live in a metro area and an apartment, respectively. |
| Schoch-Spana, 2010([17](#_ENREF_17))  N = 33  *H1N1*   - Latino (LEP) | People per Household:   - Participants reported 10-12 people or 2-3 families sharing a small cabin. |
| ***Work-related*** |  |
| Kumar, 2012([5](#_ENREF_5))  N = 2,042  *H1N1*   - AA/Black - Latino | Work-Related Inability to Social Distance:   - Controlling for age, gender, and SES, compared to Whites, Latinos were less able to social distance due to work (b = 0.37; p < 0.001). - There was no significant difference between AA/Blacks and Whites. increase in the household (b = 0.06; p < 0.05). - For each unit increase in the work-related social distancing index there was an 8% increase in the likelihood of an ILI in the participant and a 6% increase in the likelihood of an ILI in their household.   Index included: *ability to work at home, sick leave (general), sick leave for the flu, fired for no-show, job can only be done in workplace.* |
| Quinn 2011([11](#_ENREF_11))  N = 1,479  *H1N1*   - AA/Black - Latino (English and LEP) | Work-Related Inability to Social Distance:   - Controlling for age, gender, and SES, compared to Whites, Spanish-speaking Latinos were less able to social distance due to work (b = 0.89; p < 0.001). - AA/Blacks were better able to social distance (b = -0.57, p = 0.01). - There was no difference between English-speaking Latinos and Whites.   Index included: *ability to work at home, sick leave (general), sick leave for the flu, fired for no-show, job can only be done in workplace.* |
| Schoch-Spana, 2010([17](#_ENREF_17))  N = 33  *H1N1*   - Latino (LEP) | Limited Work Benefits   - Dependence on jobs with poor benefits (*eg*, sick leave, low wages) – unable to stay home when sick   School Closures and Sick Children:   - Limited ability to stay home from work when schools close or children are sick – lack of consistent childcare, children may go to work with parents. |
| ***Social Distancing*** |  |
| Kumar, 2012([5](#_ENREF_5))  N = 2,042  *H1N1*   - AA/Black - Latino | Public Transportation:   - Controlling for age, gender, and SES, Latinos were significantly more dependent on public transportation than Whites (b = 0.35; p < .01). - There was no significant difference between AA/Blacks and Whites. - There was no relationship between public transportation and self-reported ILI in the participant or household. |
| Lin, 2014,([6](#_ENREF_6)) 2018([7](#_ENREF_7))  N = 1,569  *H1N1*   - Low SES: Education   s | Social Distancing (Including Public Transportation):   - There was no difference in social distancing, staying home, or the reduction of human contact outside the household by education |
| Quinn 2011([11](#_ENREF_11))  N = 1,479  *H1N1*   - AA/Black - Latino (English and LEP) | Public Transportation:   - Controlling for age, gender, and SES, AA/Blacks (AOR = 3.0, 95% CI [1.9-4.6]), English-speaking Latinos (AOR = 2.8, 95% CI [1.5-5.3]), and Spanish-speaking Latinos (AOR = 4.0, 95% CI [2.5-6.5]) were more dependent on public transportation than Whites.   Group Childcare:   - Controlling for age, gender, and SES, AA/Blacks (AOR = 3.0, 95% CI [1.6-5.6]) and Spanish-speaking Latinos (AOR = 10.3, 95% CI [5.9-18.2]) had difficulty obtaining childcare that wasn’t with a group of children. - There was no difference between English-speaking Latinos and Whites. |
| SteelFisher 2015([12](#_ENREF_12))  N = 2,355  *H1N1*   - AA/Black - Latino - Asian - AI/AN - Low SES: Education   s | Social Distancing:   - Controlling for covariates, healthcare, and attitude, AA/Blacks, Latinos, Asians, and AI/ANs were more likely than Whites to avoid both air travel and public transportation. - Latinos and Asians, but not AA/Blacks were more likely to avoid social gatherings (no difference for AA/Blacks). - There was no difference in avoiding people with flu-like symptoms by race/ethnicity. - Controlling for covariates, healthcare, and attitude, adults with a HS diploma or less were less likely to avoid air travel and public transportation, social gatherings, and people with flu-like symptoms. |
| ***Hygiene-related Behaviors*** | |
| Lin, 2014,([6](#_ENREF_6)) 2018([7](#_ENREF_7))  N = 1,569  *H1N1*   - Low SES: Education   d | Personal Hygiene   - There was no difference in the frequency of handwashing or hand sanitizer, and no difference in coughing with mouth covered by education. |
| SteelFisher 2015([12](#_ENREF_12))  N = 2,355  *H1N1*   - AA/Black - Latino - Asian - AI/AN - Low SES: Education   s | Personal Hygiene   - Controlling for covariates, healthcare, and attitude racial/ethnic minorities were similar to or more likely than Whites to adopt hygiene-related behaviors (*eg*, covering nose and mouth, cleaning more frequently). - Adults with a HS education or less were similar to or more likely than higher educated adults to adopt hygiene-related behaviors (*eg,* covering nose and mouth, cleaning more frequently). |
| **Susceptibility** |  |
| Hennessey, 2015([19](#_ENREF_19))  N = 381  *H1N1 Mortality*   - AI/AN   D | Preexisting Conditions   - AI/ANs had higher rates of H1N1 mortality (OR = 1.95, 95% CI [1.03-3.68]). - However, preexisting conditions associated with susceptibility to H1N1 complications mediated the relationship between AI/AN race and H1N1 mortality (no data provided) |
| Levy 2013([20](#_ENREF_20))  N = 374  *H1N1 Hospitalization*   - Low SES: Education - Low SES: Neighborhood Poverty | Comorbid Conditions:   - Having 1+ comorbid conditions associated with susceptibility to H1N1 complications significantly increased the odds of hospitalization in adults (OR = 12.83, 95% CI [4.99-32.97]).   Comorbid Conditions adjusted for education and access to care:   - In multivariate analysis also including education, having a primary care provider, and health insurance, having 1+ comorbid condition significantly increased the odds of hospitalization (AOR = 7.61, 95% CI [2.68-21.65]). - After adjusting for access to care and comorbid conditions, adults with ≤ HS education (AOR = 21.21, 95% CI [5.32-84.53]) and HS graduates (vs some college or more; AOR = 3.82, 95% CI [1.64-8.90]) remained more likely to be hospitalized.   Comorbid Conditions adjusted for % neighborhood residents below FPL and access to care:   - In multivariate analysis also including having a primary care provider, health insurance, and % neighborhood below FPL, those with 1+ comorbid condition (AOR = 10.05, 95% CI [3.65-27.64]) were more likely to be hospitalized. - After adjusting for access to care and comorbid conditions, adults living in neighborhoods with 30%+ below the FPL remained more likely to be hospitalized (AOR = 5.02, 95% CI [1.83-13.89]). |
| Quinn 2011([11](#_ENREF_11))  N = 1,479  *H1N1*   - AA/Black - Latino (English and LEP)   s | Comorbid Conditions:   - Controlling for confounders, Spanish-speaking Latinos had fewer comorbidities associated with susceptibility to H1N1 complications than Whites (b = -0.37; p < 0.001). - There was no difference from Whites for English-speaking Latinos or AA/Blacks. |
| **Access to Healthcare** |  |
| Levy 2013([20](#_ENREF_20))  N = 374  *H1N1 Hospitalization*   - Low SES: Education - Low SES: Neighborhood Poverty | Public vs Private Insurance   - Adults with private insurance were less likely to be hospitalized (OR = 0.15, 95% CI [0.07-0.32]).   Primary Care Provider:   - Having a primary care provider was not significantly related to the odds of hospitalization in adults (OR = 0.88, 95% CI [0.35-2.18]).   Health Insurance:   - Having health insurance was not significantly related to the odds of hospitalization in adults (OR = 0.42, 95% CI [0.12-1.49]).   Primary Care and Health Insurance adjusted for education and comorbidities:   - In multivariate analysis that also included education, and 1+ comorbid condition, neither having a primary care provider (AOR = 1.88, 95% CI [0.50-7.05]) nor having health insurance (AOR = 0.73, 95% CI [0.14-3.70]) were related to hospitalization. ]). - Controlling for comorbidity and access to care, adults with ≤ HS education (AOR = 21.21, 95% CI [5.32-84.53]) and being a HS graduate (vs some college or more; AOR = 3.82, 95% CI [1.64-8.90]) remained more likely to be hospitalized.   Primary Care Provider and Health Insurance adjusted for % neighborhood residents below FPL and comorbidities:   - In multivariate analysis that also included % neighborhood residents below FPL and having 1+ comorbid condition, neither having a primary care provider (AOR = 1.50, 95% CI [0.42-5.30]) nor health insurance (AOR = 0.42, 95% CI [0.09-2.04]) were significantly associated with hospitalization. - Controlling for comorbidity and access to care, adults living in neighborhoods with 30%+ of residents below the FPL remained more likely to be hospitalized (AOR = 5.02, 95% CI [1.83-13.89]). |
| Quinn 2011([11](#_ENREF_11))  N = 1,479  *H1N1*   - AA/Black - Latino (English and LEP) | Access to care:   - Controlling for gender, age, and SES, Spanish-speaking Latinos had a harder time accessing care than Whites (b = 0.85, p < 0.001). - There was no significant difference between AA/Blacks and English-speaking Latinos and Whites.   Index included: *health insurance, regular provider, and the perception that lack of insurance or money would make it difficult to receive a flu shot* |
| Schoch-Spana, 2010([17](#_ENREF_17))  N = 33  *H1N1*   - Latino (LEP) | Health Centers:   - MSFW are not always aware of migrant health centers, lack transportation from rural locations   Insurance/Cost:   - Lack of money for healthcare costs, no insurance, can’t access public assistance   Limited English Proficiency   - Participants discussed the lack of Spanish and Indigenous language materials and support at health centers   Fear:   - May not seek care for fear of deportation |
| SteelFisher 2015([12](#_ENREF_12))  N = 2,355  *H1N1*   - AA/Black - Latino - Asian - AI/AN - Low SES: Education   s | Spoke to Provider about H1N1   - Controlling for covariates, healthcare, and attitude, AA/Blacks, Latinos, and AI/ANs were more likely to have spoken to a doctor or other healthcare professional about how to protect their selves or families from H1N1. - There was no difference for Asians or low SES. |
| Witrago, 2011([13](#_ENREF_13))  N = 209  *Influenza Pandemic Preparedness*   - Latino (LEP)   S | Lack of health insurance:   - The top reason (25%) for not storing/keeping medication on hand was due to lack of money or health insurance. |
| **Discrimination and Trust** |  |
| Freimuth 2014([3](#_ENREF_3))  N = 1,543  *H1N1*   - AA/Black - Latino | Trust in the government’s ability to cope with H1N1:   - More AA/Blacks and Latinos than Whites trusted the federal government (*eg*, President Obama, HHS; *p* < 0.05). - There were no differences in trust of the CDC and state and local governments by race or ethnicity   Discrimination when Seeking Health Care   - There was no difference in overall trust in government information sources among those who had experienced discrimination in health care (M = 2.18 [SE = 0.02] trust score) vs those who had not (M = 2.30 [SE = 0.07] trust score). |
| McCauley 2013([16](#_ENREF_16))  N = 46  *H1N1*   - AA/Black   s | Trust in the Government and Media:   - AA/Black - Participants in a largely AA//Black focus group expressed concerns that they didn’t feel that health experts, the government, and the media provided consistent adequate information and that they could believe. |
| Mesch, 2014([9](#_ENREF_9))  N = 968  *H1N1*   - Latino - Low SES: Education   s | Confidence in the government’s ability to cope with H1N1:   - Latinos were more likely than Whites (AOR = 1.24, 95% CI NR, *p* < 0.05) and AA/Blacks (AOR 2.39, 95% CI NR, *p* < 0.05) to trust the government’s ability to deal with H1N1 - There was no difference by education (OR = 1.00, 95% CI [0.88-1.14]). |
| Schoch-Spana, 2010([17](#_ENREF_17))  N = 33  *H1N1*   - Latino (LEP) | Community stigmatization:   - Misinformation by the media about community health centers serving migrant workers as “hotspots,” - During the H1N1 pandemic, MSFW felt they were shunned and bullied, 1 family was denied school admission despite no symptoms.   Stigmatization by Providers:   - Example: Provider overheard to say, “people [are] coming from Mexico and bringing the swine flu.” |
| Quinn 2009,([10](#_ENREF_10)) 2011([11](#_ENREF_11))  2009 N = 1,543  2011 N = 1,479  *H1N1*   - AA/Black - Latino (English and LEP)   x | Experienced Discrimination when Seeking Healthcare:   - Controlling for age, gender, and SES, AA/Blacks (AOR = 3.9, 95% CI [2.2-7.0]), English-speaking Latinos (AOR = 2.8, 95% CI [1.2-6.9]), and Spanish-speaking Latinos (AOR = 6.1, 95% CI [3.2-11.5]) were more likely than Whites to have experienced discrimination when seeking healthcare.   Trust in the government’s ability to cope with H1N1:   - Both AA/Black and Latino participants scored higher on a scale related to government trust and H1N1 (p < 0.001). |
| **Information and Knowledge** |  |
| Etingen 2012([2](#_ENREF_2))  N = 3,384  *H1N1*   - AA/Black - Latino - Low SES: Education   s | Receipt of H1N1 Information:   - Among Veterans with spinal cord injuries and disorders, report of the receipt of adequate information was more likely for Whites (vs non-Whites; OR = 1.67 [95 % CI 1.39-2.01]) and higher SES (college graduates; (OR 1.28, 95% CI [1.05-1.56]). |
| Lin, 2014,([6](#_ENREF_6)) 2018([7](#_ENREF_7))  N = 1,569  *H1N1*   - Low SES: Education   c | H1N1 Transmission Knowledge   - Participants with less education were less knowledgeable about H1N1 transmission and were more likely to avoid eating pork products. - However, both became non-significant when information barriers were considered. - There was no difference in misconceptions about H1N1 by education. |
| Lin, 2017([8](#_ENREF_8))  N = 627  *MERS and Previous Epidemics*   - AA/Black - Latino - Low SES: Education | Pandemic Awareness:   - Individuals with lower education (AOR = 3.67, 95% CI 1.44-9.36) were more likely to have a low awareness of pandemics compared with other groups. - There was no difference by race/ethnicity.   MERS Awareness:   - Compared to Whites, AA/Blacks (OR = 0.36, 95% CI [0.19–0.70], p < 0.005) and Latinos (OR = 0.46 [95% CI 0.23–0.91], p = 0.03]) were less likely to have heard of MERS. - There was no difference by SES (education).   MERS Knowledge:   - Compared to Whites, AA/Blacks (OR = 0.13, 95% CI [0.03–0.57]) were less likely to have accurate knowledge of MERS. - There was no difference between Latinos and Whites. - There was no difference by SES. |
| Witrago, 2011([13](#_ENREF_13))  N = 209  *Influenza Pandemic Preparedness*   - Rural Latinos (LEP)   v | Preparation:   - Regardless of SES, years in US, and demographics, rural Spanish-speaking Latinos were not prepared for an influenza pandemic   Index included: *available food, water, flashlight, medical supplies etc., prep plan* |
| Yip, 2009([14](#_ENREF_14))  N = 100  *H1N1*   - Chinese (LEP)   g | Informed about H1N1:   - Compared to individuals who did not speak English, those who reported not speaking English “well” were more likely to feel that they were informed about H1N1 (OR = 2.65, 95% CI [1.04-7.01], p < 0.05). |

Abbreviations: AA = African American; AI/AN = American Indian/Alaska Native; AOR = adjusted odds ratio; C = control; CDC = Centers for Disease Control; CI = confidence interval; ED = emergency department; EMR = electronic medical record; HS = high school; LEP = limited English proficiency; MERS = Middle East Respiratory Syndrome; MSFW = migrant and seasonal farmworkers; OR = odds ratio; OT = Occupational Therapy; PI = Pacific Islander; SES = socioeconomic status; US = United States

**APPENDIX REFERENCES**

1. U.S. Preventive Services Task Force. Appendix VII. Criteria for Assessing External Validity (Generalizability) of Individual Studies 2017. Available from: https://www.uspreventiveservicestaskforce.org/Page/Name/appendix-vii-criteria-for-assessing-external-validity-generalizability-of-individual-studies.

2. Etingen B, LaVela SL, Miskevics S, Goldstein B. Health information during the H1N1 influenza pandemic: Did the amount received influence infection prevention behaviors? Journal of Community Health: The Publication for Health Promotion and Disease Prevention. 2013;38(3):443-50. doi: http://dx.doi.org/10.1007/s10900-012-9647-8.

3. Freimuth VS, Musa D, Hilyard K, Quinn SC, Kim K. Trust during the early stages of the 2009 H1N1 pandemic. Journal of health communication. 2014;19(3):321-39. Epub 2013/10/11. doi: 10.1080/10810730.2013.811323. PubMed PMID: 24117390.

4. Kumar S, Quinn SC, Kim KH, Musa D, Hilyard KM, Freimuth VS. The social ecological model as a framework for determinants of 2009 H1N1 influenza vaccine uptake in the United States. Health Education & Behavior. 2012;39(2):229-43. PubMed PMID: 21984692.

5. Kumar S, Quinn SC, Kim KH, Daniel LH, Freimuth VS. The impact of workplace policies and other social factors on self-reported influenza-like illness incidence during the 2009 H1N1 pandemic. American Journal of Public Health. 2012;102(1):134-40. PubMed PMID: 22095353.

6. Lin L, Jung M, McCloud RF, Viswanath K. Media use and communication inequalities in a public health emergency: a case study of 2009-2010 pandemic influenza A virus subtype H1N1. Public Health Reports. 2014;129 Suppl 4:49-60. PubMed PMID: 25355975.

7. Lin L, McCloud RF, Jung M, Viswanath K. Facing a Health Threat in a Complex Information Environment: A National Representative Survey Examining American Adults' Behavioral Responses to the 2009/2010 A(H1N1) Pandemic. Health Educ Behav. 2018;45(1):77-89. Epub 2017/05/27. doi: 10.1177/1090198117708011. PubMed PMID: 28548547.

8. Lin L, McCloud RF, Bigman CA, Viswanath K. Tuning in and catching on? Examining the relationship between pandemic communication and awareness and knowledge of MERS in the USA. Journal of Public Health. 2017;39(2):282-9. PubMed PMID: 27084759.

9. Mesch GS, Schwirian KP. Social and political determinants of vaccine hesitancy: Lessons learned from the H1N1 pandemic of 2009-2010. American Journal of Infection Control. 2015;43(11):1161-5. PubMed PMID: 26521933.

10. Quinn SC, Kumar S, Freimuth VS, Kidwell K, Musa D. Public willingness to take a vaccine or drug under Emergency Use Authorization during the 2009 H1N1 pandemic. Biosecur Bioterror. 2009;7(3):275-90. doi: 10.1089/bsp.2009.0041. PubMed PMID: 19775200.

11. Quinn SC, Kumar S, Freimuth VS, Musa D, Casteneda-Angarita N, Kidwell K. Racial disparities in exposure, susceptibility, and access to health care in the US H1N1 influenza pandemic. American Journal of Public Health. 2011;101(2):285-93. PubMed PMID: 21164098.

12. SteelFisher GK, Blendon RJ, Kang M, Ward JR, Kahn EB, Maddox KE, et al. Adoption of preventive behaviors in response to the 2009 H1N1 influenza pandemic: a multiethnic perspective. Influenza & Other Respiratory Viruses. 2015;9(3):131-42. PubMed PMID: 25688806.

13. Witrago E, Perez MA. Preparing for an influenza pandemic: policy implications for rural Latino populations. Journal of Health Care for the Poor & Underserved. 2011;22(3 Suppl):58-71. PubMed PMID: 21857139.

14. Yip MP, Ong B, Painter I, Meischke H, Calhoun B, Tu SP. Information-seeking behaviors and response to the H1N1 outbreak in Chinese limited-English proficient individuals living in King County, Washington. American Journal of Disaster Medicine. 2009;4(6):353-60. PubMed PMID: 20104728.

15. Wells GA, Shea B, O'Connell D, Peterson J, Welch V, Losos M, et al. The Newcastle-Ottawa Scale (NOS) for assessing the quality of nonrandomised studies in meta-analyses. Available from: http://www.ohri.ca/programs/clinical_epidemiology/oxford.asp.

16. McCauley M, Minsky S, Viswanath K. The H1N1 pandemic: media frames, stigmatization and coping. BMC Public Health. 2013;13:1116. Epub 2013/12/05. doi: 10.1186/1471-2458-13-1116. PubMed PMID: 24299568; PubMed Central PMCID: PMCPMC3907032.

17. Schoch-Spana M, Bouri N, Rambhia KJ, Norwood A. Stigma, health disparities, and the 2009 H1N1 influenza pandemic: how to protect Latino farmworkers in future health emergencies. Biosecurity & Bioterrorism. 2010;8(3):243-54. PubMed PMID: 20825335.

18. Critical Appraisal Skills Programme. CASP Qualitative Checklist 2018 [27 May 2020]. Available from: https://casp-uk.net/wp-content/uploads/2018/01/CASP-Qualitative-Checklist-2018.pdf.

19. Hennessy TW, Bruden D, Castrodale L, Komatsu K, Erhart LM, Thompson D, et al. A case-control study of risk factors for death from 2009 pandemic influenza A(H1N1): is American Indian racial status an independent risk factor? Epidemiology & Infection. 2016;144(2):315-24. PubMed PMID: 26118767.

20. Levy NS, Nguyen TQ, Westheimer E, Layton M. Disparities in the severity of influenza illness: a descriptive study of hospitalized and nonhospitalized novel H1N1 influenza-positive patients in New York City: 2009-2010 influenza season. Journal of Public Health Management & Practice. 2013;19(1):16-24. PubMed PMID: 23169399.
